# The complete defensive strategy: Defining complementary pitching and fielder positioning strategies in baseball

**DOI:** 10.1371/journal.pone.0341045

**Published:** 2026-10-07

**Authors:** William Melville, Christopher Archibald, David Grimsman

**Affiliations:** Department of Computer Science, Brigham Young University, Provo, Utah, United States of America; Istanbul Technical University: Istanbul Teknik Universitesi, TÜRKIYE

## Abstract

In baseball, the characteristics of a pitch have an effect on a batted ball’s trajectory. For example, batters are more likely to hit a fastball to the opposite side of the field and to pull a changeup. In this paper, we present novel matchup and pitch-specific approaches to positioning fielders to maximize outs and minimize runs allowed against opposing hitters. Unfortunately, the pitch-specific approach may reveal information about the upcoming pitch to the opposing batter. Thus, we combine our pitch-specific positioning strategy with an existing pitch selection game theory model that assumes the batter can observe the pitch up to a decision point, at which point they must decide whether to swing at or take the pitch. The combined pitching and positioning strategies, which we call the complete defensive strategy, complement each other by mixing in an occasional bluff where the pitcher throws the wrong pitch for the positioning strategy. Thus, the batter cannot use the positioning strategy to perfectly predict the upcoming pitch, and the defense is able to utilize the pitch-specific positioning strategy. We show that the matchup and pitch-specific approaches to positioning are not significantly more effective at preventing hits or runs than a previously defined batter-specific approach. Thus, although a baseball team could use our methodology to define pitch-specific positioning strategies that do not tip the pitch to the batter, they could define similarly effective positioning strategies using much simpler models that only consider the batter’s tendencies.

## 1. Introduction

In baseball, it is the responsibility of a team’s defense to prevent runs by getting opposing hitters out. One prominent strategic decision a defense must make is how to position their seven fielders behind the pitcher and catcher. Fielder positioning strategy has been thoroughly researched [1–7]. One common criticism is that the positioning strategy and pitching strategy are defined separately when really they should complement each other. Fig 1 shows the distribution of Major League Baseball (MLB) batted ball spray angles and launch angles from 2019 through 2023 [8–10]. Typically a spray angle of −45 corresponds to the third baseline and 45 corresponds to the first baseline, but we flipped the sign of the spray angles for left-handed batters so we could compare them directly with right-handed batters. Thus, a *pulled* batted ball has a negative spray angle and an *opposite* field batted ball (oppo) has a positive spray angle. It is apparent that hitters generally pull non-fastballs more than fastballs, and they hit low pitches with lower launch angles than high pitches. It follows that if a pitcher plans to attack a hitter with breaking balls below the strike zone, a complementary positioning strategy may set infielders on the pull side of the infield to field pulled ground balls. Similarly, if a pitcher plans to attack a hitter with elevated fastballs, a complementary positioning strategy may shade outfielders to the opposite side to field oppo fly balls. Even when the positioning strategy is defined separately from the pitching plan, it may still benefit from considering the pitcher’s repertoire. For example, it may be optimal to shade fielders more oppo for a pitcher who throws 98 mph fastballs than for a pitcher who throws 88 mph fastballs whether they plan to attack the opposing hitter with fastballs or not.

**Fig 1:**
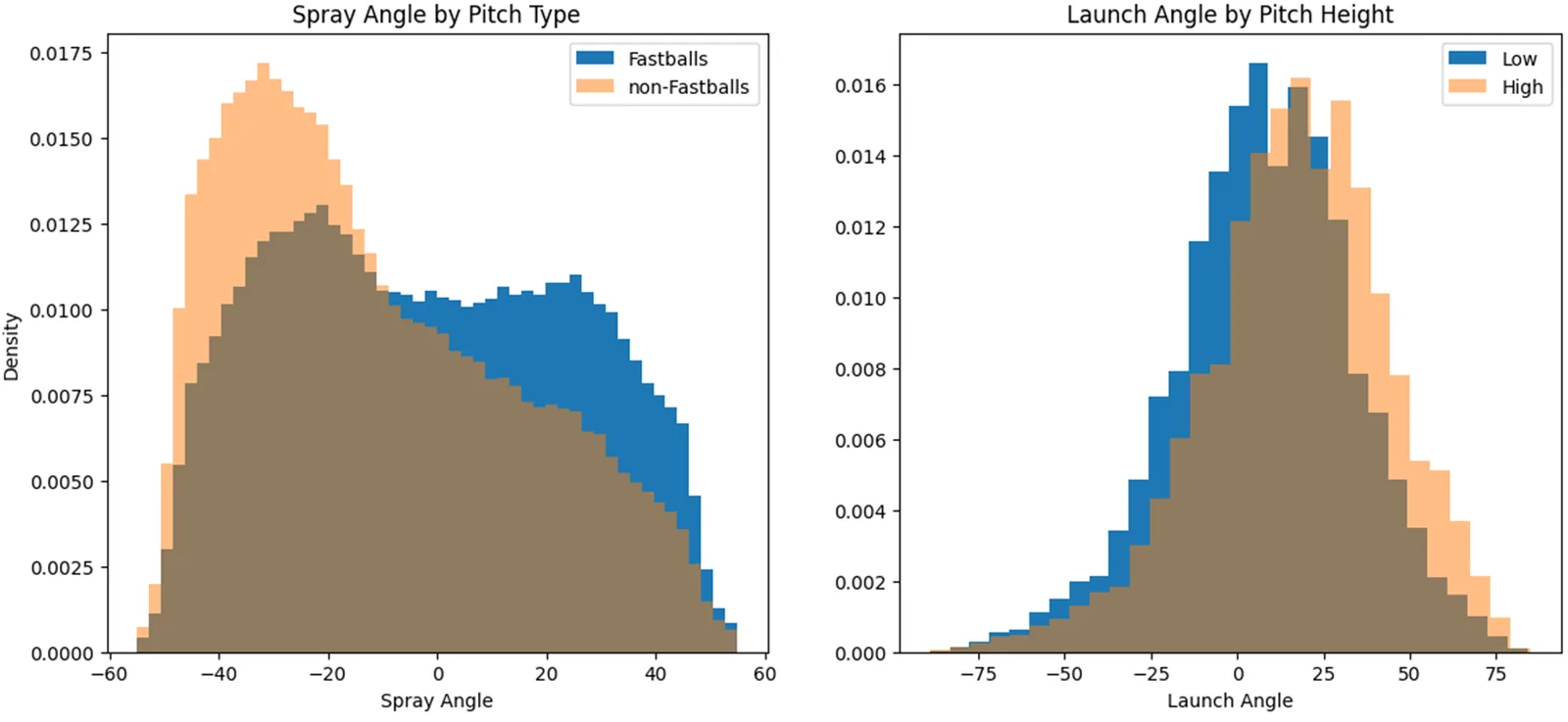
MLB spray and launch angles. Distribution of MLB spray angles partitioned by pitch type and launch angles partitioned by pitch height. It is evident that batters tend to pull non-fastballs more than fastballs and tend to hit low pitches lower than high pitches.

In this paper, we address the concern that positioning strategies do not appropriately complement pitchers and their pitching strategies by adapting the batter-specific positioning model of [7] to define pitcher and pitch-specific positioning strategies. In particular, we make four key contributions:

1 A novel model of batted ball trajectory probability distributions that is made up of three density neural networks [11]. These neural networks consider both batter tendencies and the effect that pitches have on batted balls, and they are more accurate than the batter-only models of [7].
2 A matchup (pitcher and batter) specific positioning strategy that prevents more runs and hits than the batter-specific positioning strategy of [7].
3 A pitch-specific positioning strategy that prevents more runs and hits than the matchup-specific positioning strategy as well as the batter-specific strategy of [7].

Of course, a team may hesitate to adopt the pitch-specific positioning strategy since using a different positioning strategy for every pitch would tip the pitch to the batter. Rather than choosing a pitch and then the corresponding pitch-specific positioning strategy, the pitching strategy and positioning strategy should be defined so as to ensure that the positioning strategy does not reveal too much information about the upcoming pitch and to ensure that the pitcher throws pitches that if hit in play will result in batted balls that the fielders are in a good position to field. Thus, in Section 2.3 we present the fourth and final contribution of this paper:

4 A complete defensive strategy defined by complementary positioning and pitching strategies.

In [7], they define an optimal positioning strategy as one that minimizes the expected runs allowed over a simulation of batted balls, i.e., a $\psi^{*}$ satisfying


$$\begin{array}{c} \psi^{*}\in \underset{\psi \in \Psi }{\text{arg min}}{𝔼}_{𝐛\sim d}[f(𝐛,\psi )] \end{array}$$ (1)

where Ψ is the set of admissable positioning strategies, 𝐛 is a random variable of batted ball trajectories drawn from a batter-specific batted ball trajectory probability distribution *d*, and *f* is the objective function representing expected runs allowed. We adapt this model to define pitcher and pitch-specific positioning strategies by replacing *d* with our pitch-specific batted ball trajectory density neural networks (Section 2.1).

The complementary pitching and positioning strategies that make up the complete defensive strategy are given by an equilibrium in a zero-sum game where the defense chooses the positioning strategy and pitching strategy to minimize expected runs, and the batter chooses their swing-decision strategy to maximize expected runs. An equilibrium is a positioning strategy $\psi^{*}$, pitching strategy $p^{*}$, and batter swing decision strategy $s^{*}$ satisfying


$$\begin{array}{c} \psi^{*},p^{*}\in \underset{\begin{array}{c} p\in \Delta P\\ \psi \in \Psi \end{array}}{\text{arg min}}\max_{s\in [\Delta S]}U(p,s,c,\psi ) \end{array}$$ (2a)

$$\begin{array}{c} s^{*}\in \underset{s\in [\Delta S]}{\text{arg max}}\min_{\begin{array}{c} p\in \Delta P\\ \psi \in \Psi \end{array}}U(p,s,c,\psi ), \end{array}$$ (2b)

where Ψ is again the set of admissable positioning strategies, ΔP is the set of pitching strategies, [ΔS] is the set of batter swing-decision strategies, *c* is the game context which is defined by the batter, pitcher, and ball and strike count, and *U* is the utility function that outputs expected runs given a defensive strategy, batter strategy, and game context. We describe ΔP, [ΔS], and *U* in more detail in Section 2.3. A key difference between (1) and (2) is in (1) the batter does not choose a counterstrategy to the defense’s positioning strategy ψ, but in (2) the batter chooses their counterstrategy *s* to maximize utility against the defense’s positioning and pitching strategies. This difference requires different objective functions, hence why in (1) the objective function *f* is a function of just the batted ball and the positioning strategy, but in (2) the objective function *U* is a function of the pitching strategy, positioning strategy, and batter strategy. The game model in (2) allows us to utilize the pitch-specific positioning strategies without completely revealing the upcoming pitch to the batter.

The matchup-specific positioning strategy and the complete defensive strategy effectively address the criticism that positioning strategies should complement the pitcher or the pitching strategy. However, we find that these approaches are much more complex than a batter-specific approach, and they do not result in significantly fewer hits or runs allowed (Table 1). We therefore conclude that baseball teams that wish to prioritize simplicity with a positioning strategy that only considers the batters’ tendencies are justified in doing so, and they will not necessarily have a significantly worse defense than teams with more complex, pitcher or pitch-specific positioning strategies.

**Table 1:** Comparison of the positioning strategies.

Positioning	xBA	xBA p value	xHits	xwOBA	xwOBA p value	xwRAA
Batter Specific	0.305		623	0.308		−16
Matchup Specific	0.304	0.238	621	0.308	0.32	−18
Pitch Specific	0.303	0.04	617	0.306	0.10	−20

## 2. Methods and results

### 2.1. The batted ball trajectory neural networks

Denote *h*, *v*, and ϵ as the horizontal spray angle, vertical launch angle, and exit speed of a batted ball. Our goal is to model the joint distribution 𝒫(h,v,ϵ). We learn this model using MLB ball tracking data, which provides the exit speed, launch angle, and hit coordinates of batted balls [8–10]. We define the spray angles using the hit coordinates with the formula defined by [12], which gives balls hit down the third baseline an angle of $-45^{\circ }$ and balls hit down the first baseline an angle of $45^{\circ }$. By the chain rule of probability, we can write the joint distribution 𝒫(h,v,ϵ) as


$$\begin{array}{c} 𝒫(h,v,\epsilon )=𝒫(h)𝒫(v|h)𝒫(\epsilon |v,h). \end{array}$$ (3)

Thus, we model the joint distribution using three density neural networks. First, we train a neural network to model the spray angle distribution 𝒫(h), then we train a neural network to model the launch angle distribution conditioned on spray angle 𝒫(v|h), and finally we train a neural network to model the exit speed distribution conditioned on both launch and spray angle 𝒫(ϵ|v,h).

#### 2.1.1. The spray angle distribution 𝒫(h).

We prepare the data by first flipping the sign of spray angles for left-handed batters, as was done in Fig 1, which transforms them into right-handed batters. This gives all pulled balls negative spray angles and all oppo balls positive spray angles. Information can now be shared among all hitters without effects from handedness. We then remove spray angles outside of the interval [−55,55]. Finally, we shift and scale the spray angles so they are between 0 and 1, which allows us to learn Beta distributions which have support [0,1].

The spray angle density neural network models the shifted and scaled spray angle distribution using a mixture of Betas. Each batter is represented by an index, which is first passed through an embedding layer that outputs a 15-dimensional batter embedding vector. This embedding vector is concatenated with the other inputs, which are the batter and pitcher handedness and the z-scores of a pitch’s release speed, horizontal and vertical movement, and horizontal and vertical plate location. The batter embedding ensures that the resulting spray angle distribution is batter specific, and the pitch characteristics ensure that it is pitch specific. The concatenated vector is passed through a feed forward network made up of four linear layers with ReLU activation functions between them. The output of the feed forward network is passed through three separate components: the weights component, the α component, and the β component. The weights component is a linear layer with a softmax activation to ensure the weights sum to one. The α component is a linear layer whose output is passed through a ReLU activation function and finally added to [1,1]. The β component is a linear layer whose output is passed through a ReLU activation function and finally added to [1,1]. The purpose of adding the ReLU outputs to [1,1] is to ensure that the α and β parameters in the Beta distributions are positive. Fig 2 depicts the spray angle neural network architecture. The network was trained to minimize the average negative log likelihood of the spray angle data in the output distribution.

**Fig 2:**
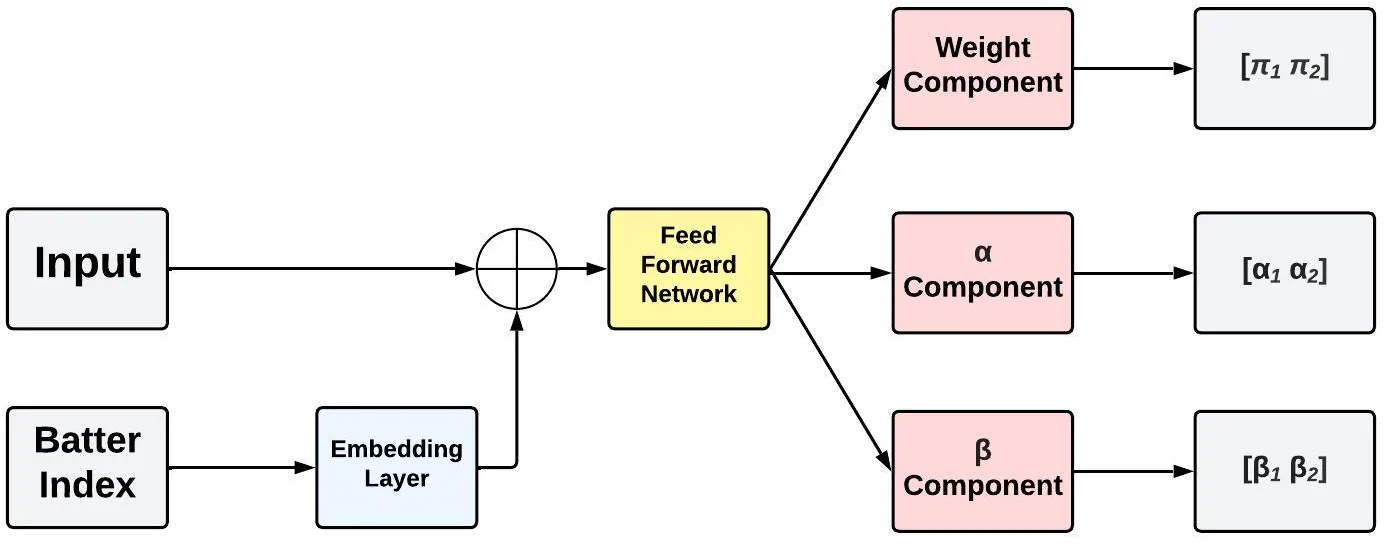
𝒫(h) neural network. This takes as input a pitch and a batter and outputs a spray angle distribution that is batter and pitch-specific.

We trained the neural network on all MLB batters’ spray angle data from 2020-2022 and validated on data from 2023. For each batted ball in the validation set, we sampled a spray angle from the corresponding neural network spray angle distribution. The results are shown in Fig 3. The plot on the left shows the actual spray angles in the validation set and the plot on the right shows the samples drawn using the neural net. The neural net learned that different pitch types tend to have different spray angles, as we observed in Fig 1. It also fits the validation data better than the spray angle model from [7] that only considers the batters’ tendencies. The log likelihood of the neural net on the validation data was 0.178, whereas the log likelihood of the batter-only model was 0.104.

**Fig 3:**
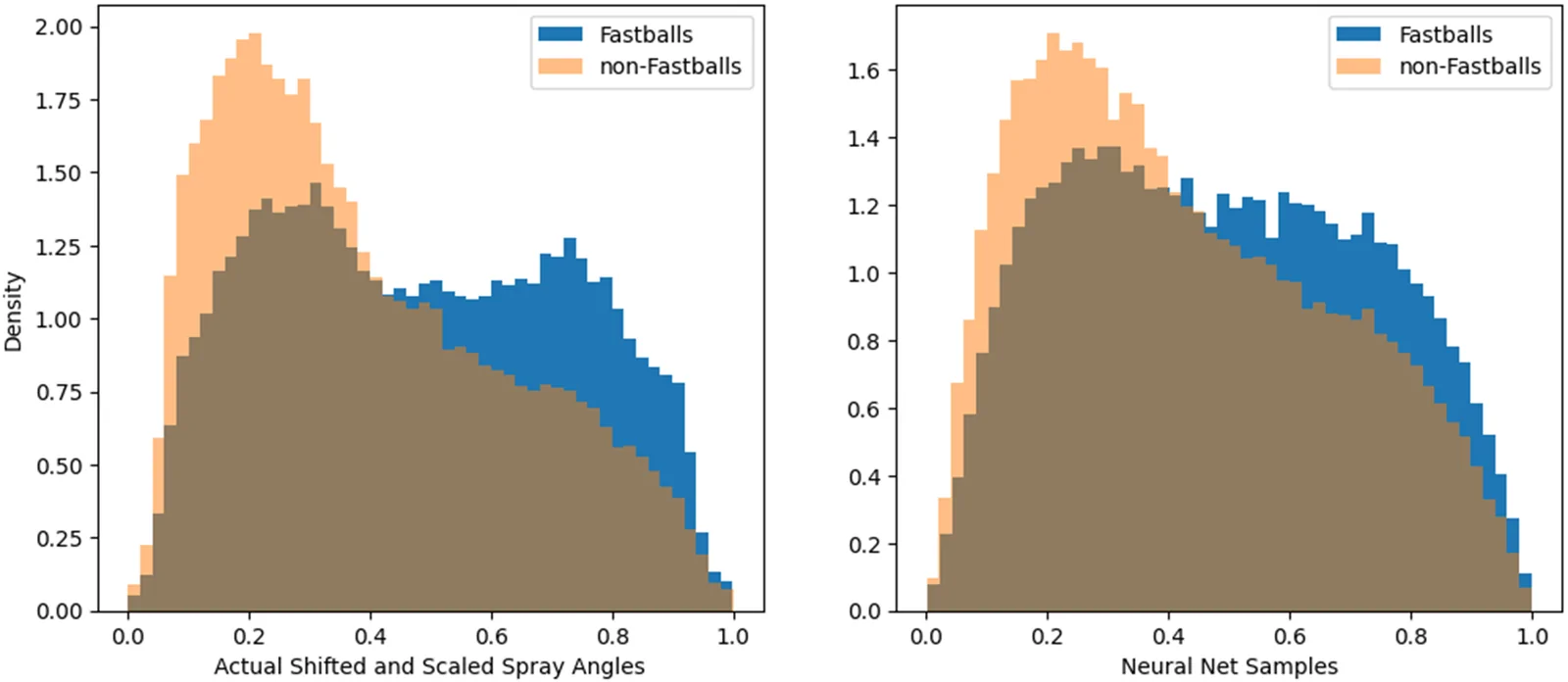
Spray angle samples. Spray angles in the validation data compared to spray angle samples from the neural net. The neural net learns to differentiate between fastballs and non-fastballs, similar to the actual spray angles.

The spray angle neural network also learned the different tendencies between batters. Fig 4 shows the actual spray angles in both the training and validation datasets of left-handed batters Nathaniel Lowe and Joey Gallo compared with the spray angles sampled by the neural net. Gallo is famously very pull heavy, whereas Lowe tends to hit more up the middle and oppo, and the neural network was able to learn that distinction.

**Fig 4:**
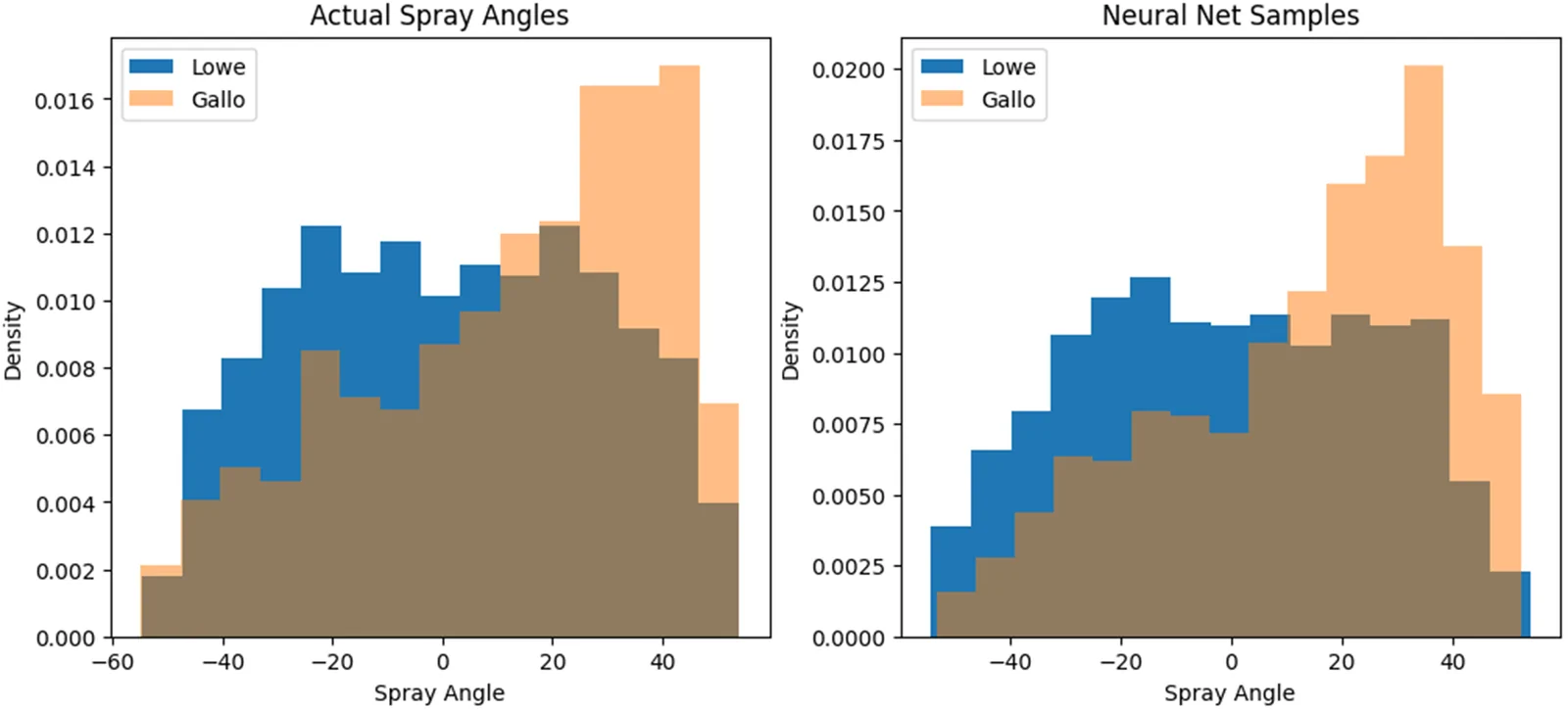
Gallo vs. Lowe. Actual spray angles compared with sampled spray angles for Joey Gallo and Nathaniel Lowe. The neural network was able to learn that Gallo is very pull heavy and Lowe tends to go more up the middle and oppo.

#### 2.1.2. The launch angle conditional distribution 𝒫(v|h).

The launch angle neural network uses the same inputs as the spray angle network as well as the z-scored spray angle (with sign flipped for left-handed batters), which satisfies our requirement that our launch angle distribution is conditioned on spray angle. The launch angle network architecture is similar to the spray angle network. First, the batter index is passed through an embedding layer, which outputs a 15-dimensional batter embedding. That is concatenated back onto the other inputs, and the resulting vector is passed through a feed forward network with four linear layers with ReLU activation functions between them. The output of the feed forward network is passed through a mean component and a standard deviation component. The mean component is just a linear layer, and the standard deviation component is a linear layer whose output is passed through a ReLU activation function and then added to one. The outputted mean and standard deviation define a Normal distribution. The launch angle network is depicted in Fig 5.

**Fig 5:**
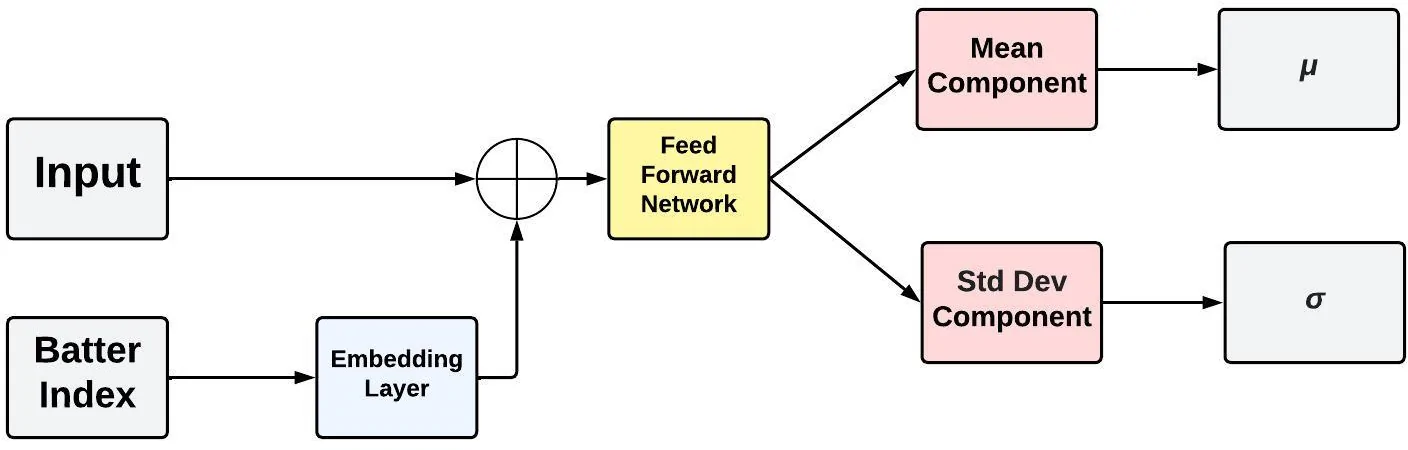
𝒫(v|h) neural network. This takes as input a pitch, a batter, and a spray angle and outputs a batter and pitch-specific launch angle Normal distribution conditioned on spray angle.

The launch angle network was trained to minimize the average negative log likelihood of the launch angle data in the output Normal distribution. Then for each batted ball in the validation set, we sampled a launch angle from the neural net. The results are shown in Fig 6 where we see that the neural net samples lower launch angles for low pitches and higher launch angles for high pitches, similar to the actual launch angle data. Fig 7 shows that the neural net was also able to learn different tendencies between batters. Joey Gallo is more of a fly ball hitter, and Yandy Diaz is more of a ground ball hitter. The neural net correctly samples higher launch angles for Gallo than Diaz.

**Fig 6:**
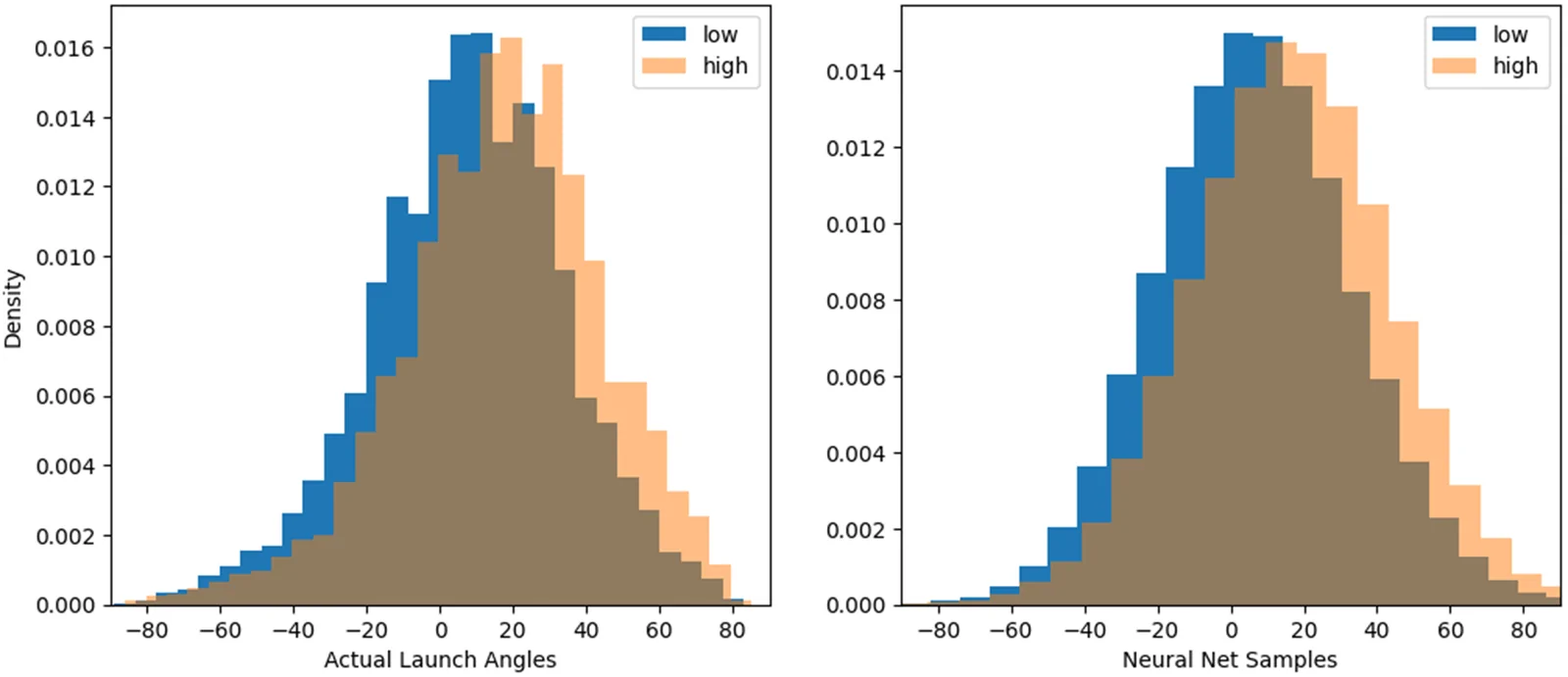
Launch angle samples. Launch angles in the validation data compared to launch angle samples from the neural net. The neural net samples differentiate between low and high pitches, similar to the actual launch angles.

**Fig 7:**
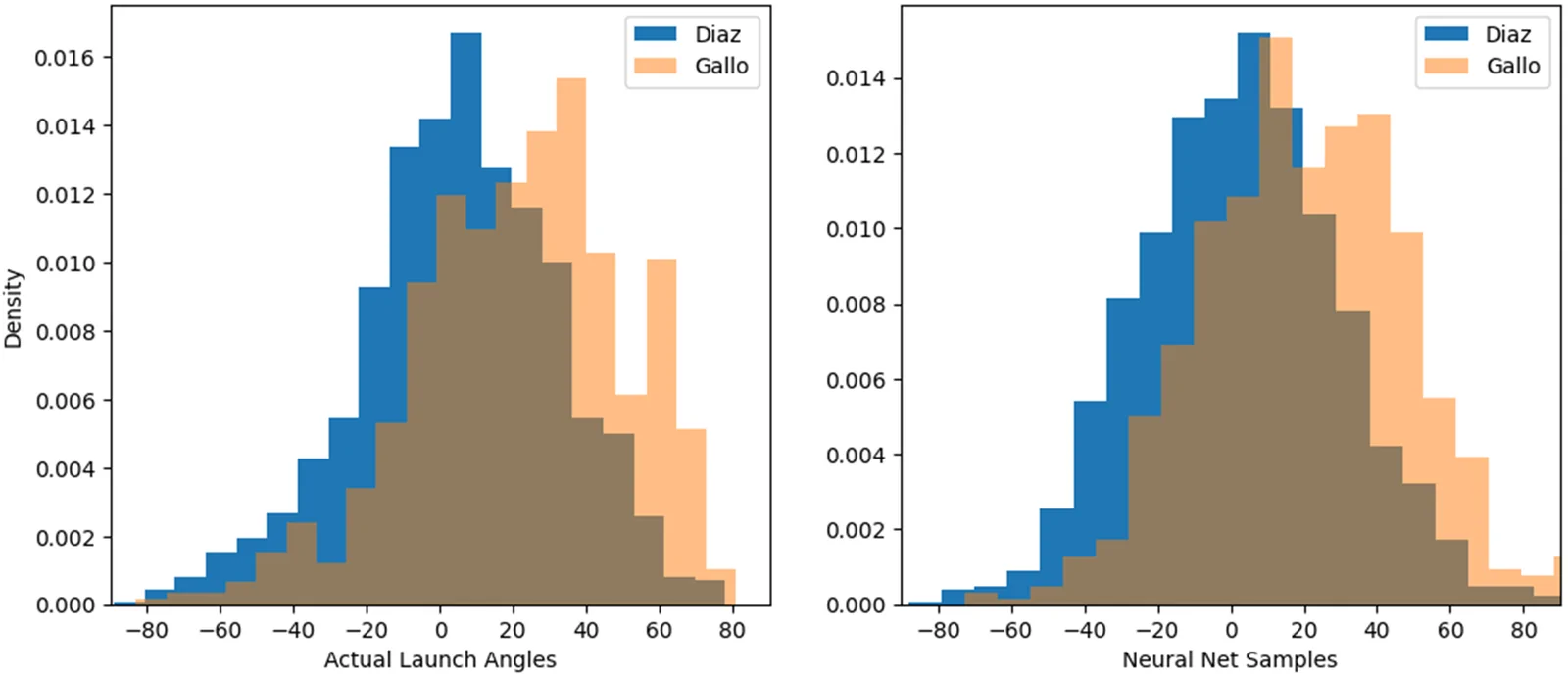
Gallo vs. Diaz. Actual launch angles compared with sampled launch angles for Joey Gallo and Yandy Diaz. The neural network was able to learn that Gallo is more of a fly ball hitter and Diaz is more of a ground ball hitter.

Lastly, just like with the spray angle network, we compared the launch angle network with the launch angle model in [7] that only considers the batters’ tendencies. The log likelihood on the validation data for the neural network was −4.54, which is better than the batter-only model’s log likelihood of −4.67.

#### 2.1.3. The exit speed conditional distribution 𝒫(ϵ|v,h).

Exit speeds are left skewed, but we can transform them into a right skewed distribution through the transformation z=max(ϵ)−ϵ. Thus, our exit speed neural network outputs a Gamma distribution that is trained to minimize the negative log likelihood of the transformed variable *z* because Gamma distributions have support (0,∞) and can fit right-skewed data. The exit speed network uses the same inputs as the launch angle network, including the z-scored spray angle, as well as the z-scored launch angle. As in the other two networks, the batter index is first passed through an embedding layer, which outputs a 15-dimensional batter embedding that is then concatenated back onto the other inputs. The resulting vector is passed through two separate components: the shape component and the rate component. These components have identical structure. They are made up of three linear layers with ReLU activation functions between them and then one final linear layer who’s output is passed through an ELU activation function and then added to one. This ensures that the outputs are positive, as required by the shape and rate parameters in a Gamma distribution. The network architecture is shown in Fig 8.

**Fig 8:**
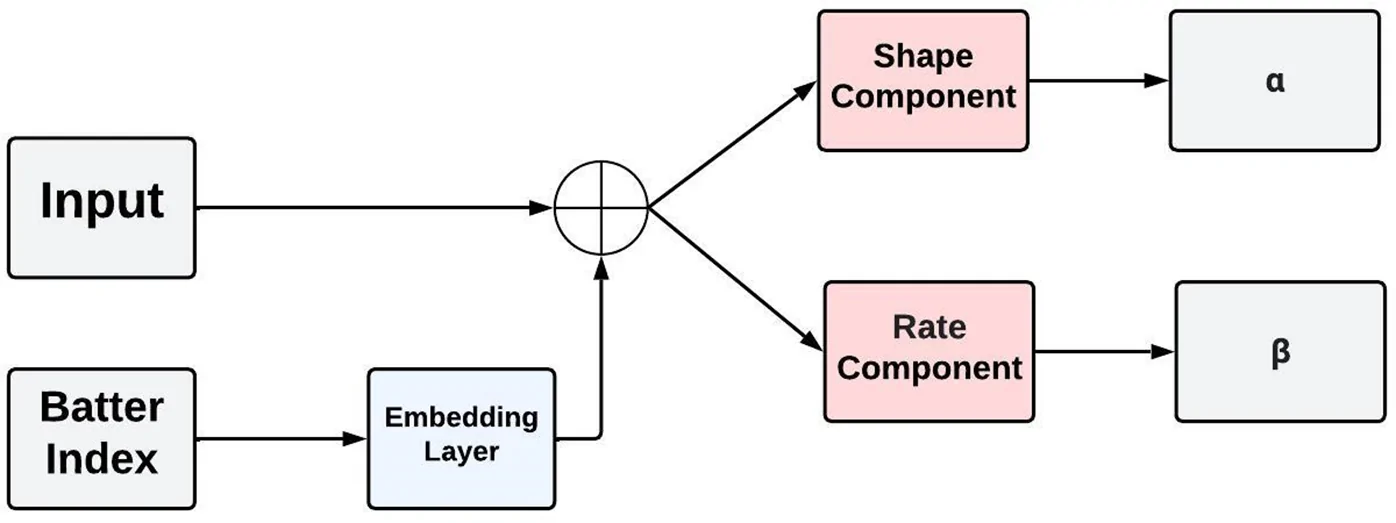
𝒫(ϵ|v,h) neural network. This takes as input a pitch, a batter, a spray angle, and a launch angle and outputs a batter and pitch-specific exit speed Gamma distribution conditioned on spray angle and launch angle.

The exit speed network was trained to minimize the negative log likelihood of the exit speed data in the output Gamma distribution. Then for each batted ball in the validation set, we sampled an exit speed from the neural network. The results are shown in Fig 9, which shows that the network generates reasonable exit speed samples. Fig 10 compares the actual and sampled exit speeds for hard-hitting Yordan Alvarez and weak-hitting Nicky Lopez. The neural network correctly learned to generate weaker exit speeds for Lopez than for Alvarez. Finally, the exit speed neural network had a better log likelihood of −3.63 on the validation data than the batter-only exit speed model of [7], which had a log likelihood of −3.94.

**Fig 9:**
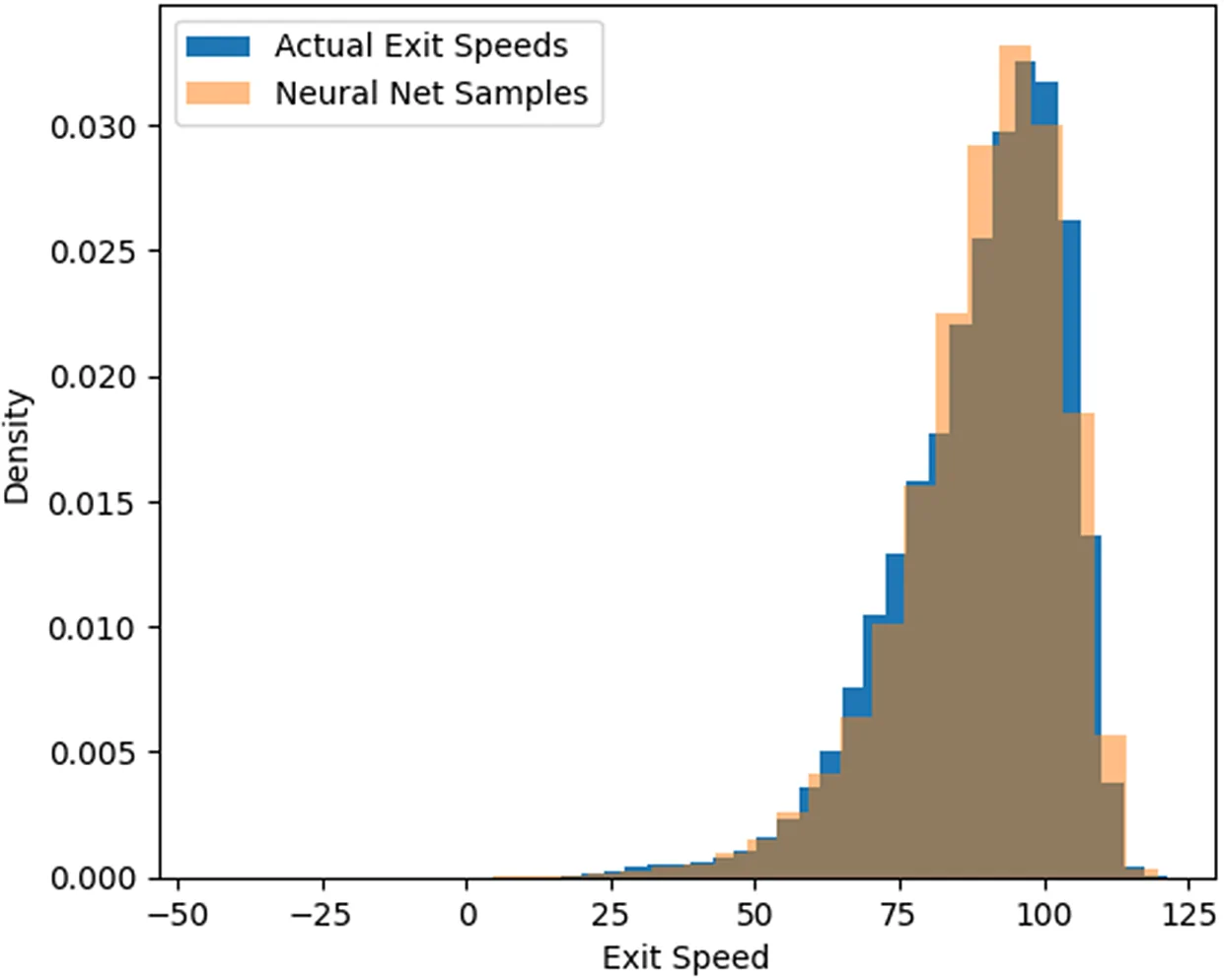
Exit speed samples. Exit speeds in the validation data compared to exit speed samples from the neural net.

**Fig 10:**
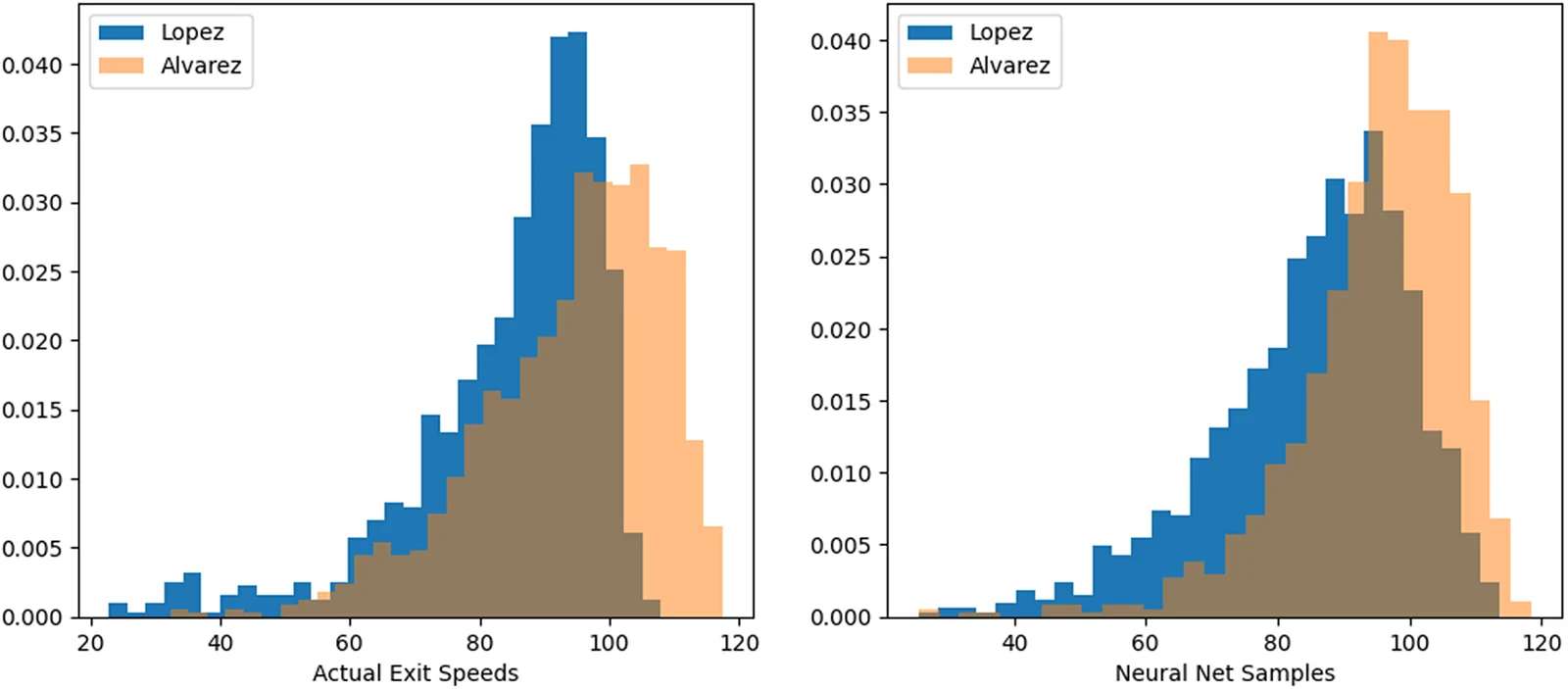
Alvarez vs. Lopez. Actual and sampled exit speeds for hard-hitting Yordan Alvarez compared to weak-hitting Nicky Lopez. The neural network correctly generates lower exit speeds for Lopez.

### 2.2. Defining and evaluating optimal positioning strategies

Recall, we define an optimal positioning strategy as a $\psi^{*}$ satisfying Eq (1),


$$\begin{array}{c} \psi^{*}\in \underset{\psi \in \Psi }{\text{arg min}}{𝔼}_{𝐛\sim d}[f(𝐛,\psi )]. \end{array}$$

[7] defined two different objective functions: *f*_outs_ and *f*_woba_. *f*_outs_ represents the negative expected outs, so minimizing it is equivalent to maximizing expected outs. *f*_woba_ represents the expected weighted on base average (wOBA). wOBA is related to runs by an affine transformation [13], so minimizing *f*_woba_ is equivalent to minimizing runs. *f*_outs_ and *f*_woba_ are defined in more detail in Appendix A. We defined our positioning strategies by minimizing *f*_woba_.

Like [7], we find an approximately optimal solution to (1) using stochastic gradient descent with momentum. Stochastic gradient descent is an unconstrained optimization method, however, the positioning strategies ψ are constrained to be in the set of admissable positioning strategies Ψ. To meet the constraints created by the 2023 shift limits [14], we use projected stochastic gradient descent [15]. After each step of gradient descent we check that three conditions are met:

1. The first baseman must be within 40 feet of first base. If not, his position is projected back into Ψ by maintaining the relative angle with first base while decreasing the distance back down to 40 feet.
2. Every infielder must be in the infield dirt, i.e., they must be within 95 feet of the pitching rubber [16]. If not, their position is projected back into Ψ by maintaining the relative angle with the rubber while decreasing the distance back down to 95 feet.
3. There must be two infielders on either side of second base. If the third baseman or shortstop have spray angles greater than $0^{\circ }$, they are projected back into Ψ by maintaining the depth from home plate while decreasing the spray angle back down to $0^{\circ }$. Likewise, if the second baseman has a spray angle less than $0^{\circ }$, his position is projected back into Ψ by maintaining the depth from home plate while increasing the spray angle back up to $0^{\circ }$.

The difference between the batter-specific positioning strategies of [7] and our novel matchup and pitch-specific positioning strategies is the choice of batted ball trajectory distribution *d* used to simulate the batted balls 𝐛. For the matchup-specific positioning strategies, we start by taking all available pitch tracking data where the pitcher in the matchup faced a batter of the same handedness as the batter in the matchup. For example, to find a positioning strategy when right-handed pitcher Nick Anderson faces right-handed batter Mookie Betts, we start with all the pitches that Anderson threw to any right-handed batter. The resulting set of pitches are run through a machine learning model that predicts the probability of a ball in play given the pitch characteristics and the handedness of the batter and pitcher. If $p_{i}$ is the predicted probability of a ball in play on the *i*^th^ pitch in the data, then we choose to keep pitch *i* in our dataset with probability $p_{i}$ and remove it with probability $1-p_{i}$. Finally, we sample some number of pitches with replacement from the remaining dataset. The resulting sample represents a set of pitches that the pitcher in the matchup might realistically throw to the batter in the matchup and that the batter in the matchup could realistically hit into play. We finally run each pitch as well as the batter’s index through our density neural networks from Section 2.1, and we sample one batted ball from each of the resulting joint probability distributions over spray angle, launch angle, and exit speed. We use the exact same approach for our pitch-specific positioning strategies only we filter the initial pitch dataset down to only include the pitch type of interest. For example, to find a positioning strategy for Anderson’s curveball against Betts, the initial pitch dataset would be all the curveballs that Anderson threw to a right-handed batter.

When drawing batted ball samples, we use Tropicana Field’s outfield dimensions, acquired from [17], to identify batted balls that would land foul or over the Trop’s outfield fence. Tropicana Field was chosen because it is a dome; its atmospheric conditions are consistent. Thus, we can more consistently estimate the landing spot and hang time of a batted ball given its spray angle, launch angle, and exit speed assuming it was hit at the Trop rather than an outdoor stadium with inconsistent weather conditions. Nevertheless, it is straightforward to estimate the landing spot and hang time models in more extreme, outdoor environments for baseball teams who are not playing indoors [7].

To test the effectiveness of our matchup and pitch-specific positioning strategies compared to [7]’s batter-specific positioning strategy, we used *f*_outs_ and *f*_woba_ to calculate the expected outs and wOBA of each positioning strategy on 61,200 batted balls in 2023 that were not home runs and that would have stayed in Tropicana Field. To give an idea of the effect that the pitch has on positioning, Fig 11 gives a histogram of the distance between the batter-specific positioning and the pitch-specific positioning on each of the 61,200 batted balls broken down by the outfield, middle infield (shortstop and second base), and corner infield (first and third base). To get from the batter-specific positioning to the pitch-specific positioning, a corner infielder has to move an average of 5 feet, a middle infielder has to move an average of 9.5 feet, and an outfielder has to move an average of 11 feet, so the pitch has a noticeable effect on the recommended positioning.

**Fig 11:**
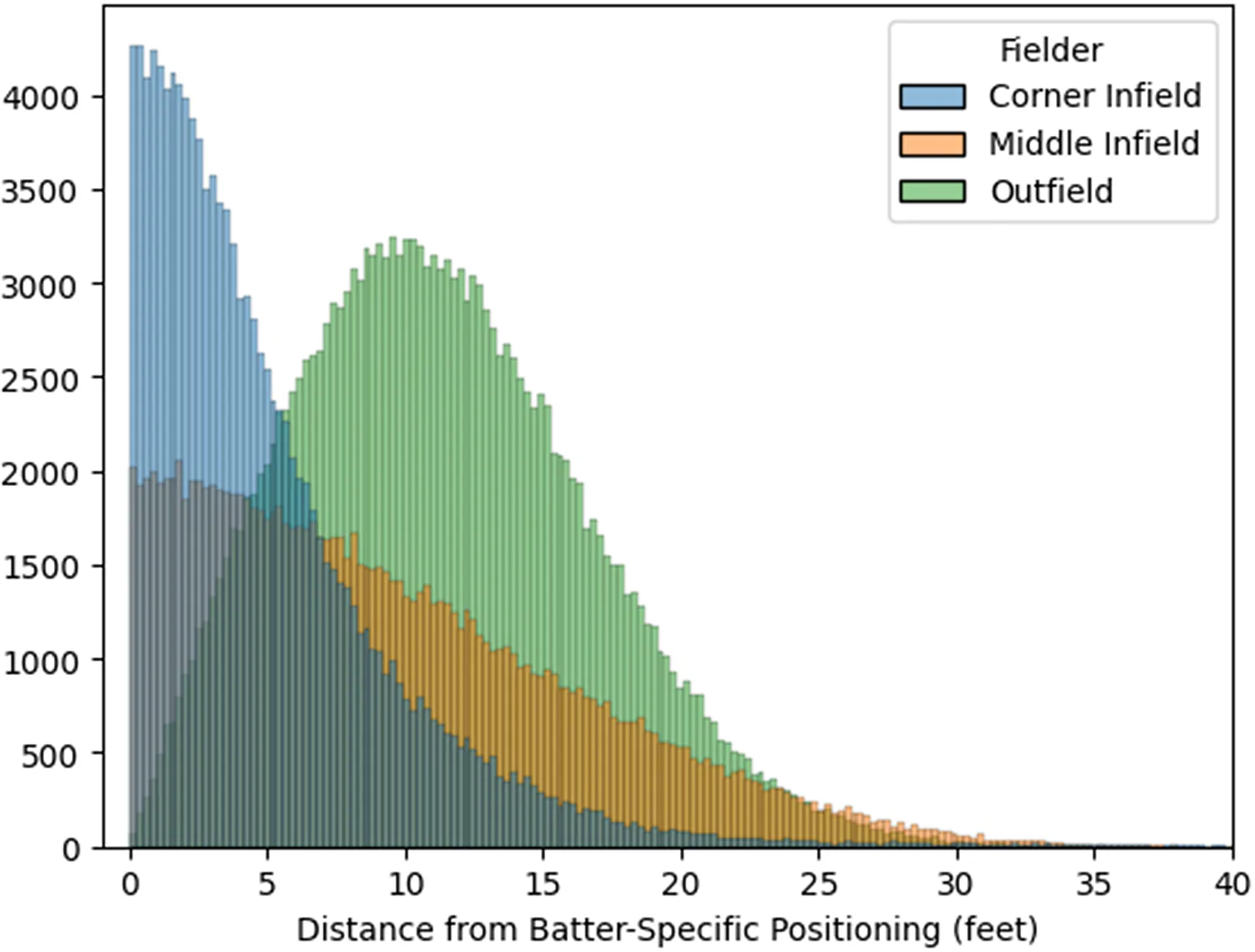
Histograms giving the distance that a corner infielder, middle infielder, or outfielder has to travel to get from the batter-specific positioning to the pitch-specific positioning. All fielders will change their positioning by a noticeable amount when the pitch is incorporated into the positioning strategy, with outfielders moving the furthest, followed by middle infielders, and then corner infielders moving the least.

Table 1 shows the expected batting average, the expected hits allowed per team, the expected wOBA, and the expected weighted runs above average (wRAA) per team of each positioning strategy on the 61,200 batted balls. Note that wRAA was estimated by applying the affine transformation to wOBA to get runs [13]. Table 1 also reports p values in two-sample t-tests comparing the mean expected batting averages and expected wOBAs of the matchup and pitch-specific positioning strategies with the means of the batter-specific strategies. The alternative hypotheses in those t-tests are that the matchup and pitch-specific means are less than the batter-specific means.

The results show that all three positioning strategies effectively prevent runs compared to MLB average, as measured by wRAA. The pitch-specific positioning strategy is the best at preventing runs and hits, followed by the matchup-specific positioning strategy. However, none of the improvements over the batter-specific positioning strategy are statistically significant at a significance threshold of α=0.05 except for the pitch-specific strategy’s improvement to expected batting average. Even that is only worth two fewer hits per one thousand batted balls. This result partially contradicts the common criticism that effective positioning strategies must complement the pitching strategy or the pitcher’s repertoire. It seems that defenses can create effective positioning strategies that only consider the batter’s tendencies. Nevertheless, some teams who are desperate enough for a small competitive advantage may be willing to take on the added complexity of the pitch-specific positioning approach. Thus, in the next section we present our complete defensive strategy, which allows defenses to use the pitch-specific strategies without tipping their pitch to the batter.

### 2.3. The complete defensive strategy

A complete defensive strategy must consider the interplay between the positioning, pitching, and batter swing-decision strategies and their effect on the expected run value of a matchup between batter and defense. That is why we propose the zero-sum batter/defense game model with equilibrium (2) as an appropriate solution to identifying a complete defensive strategy. This game is largely based on the pitching game from [18], which we review in the following subsection. We then show how to incorporate the positioning strategy into this pitching game to get our complete defensive strategy. We conclude with an example implementation of our complete defensive strategy.

#### 2.3.1. The pitching game.

This subsection serves as a review of the pitching game of [18]. The pitching game is a zero-sum game between pitcher and batter where the batter has to choose whether or not to swing at a pitch based on the information they can observe about the pitch. Therefore, each pitch can be partitioned into some properties that are observable and some that are unobservable to the batter. Denote the set of possible observable properties as $P_{o}$ and the set of possible unobservable properties as $P_{u}$. The pitcher takes action by choosing an intended pitch from the action set $P=P_{o}\times P_{u}$. It is important to note that the pitcher’s action is an *intended* pitch not an actual pitch. Pitchers do not always hit their intended target, so [18] developed a model of pitcher command skill which they used to estimate the probability distribution over actual pitches in *P* given a choice of intended pitch in *P*.

The pitcher’s pitching strategy is the decision rule that they use to choose an action in *P*. The pitcher is allowed to use a *behavioral strategy*, which is a probability distribution over all of the possible actions in *P*. The pitcher takes action under a behavioral strategy by randomly sampling an intended pitch from the behavioral strategy distribution. It is important to allow the pitcher to use a behavioral strategy because it allows them to take advantage of the uncertainty the batter has about the incoming pitch due to the unobservable properties. If the pitcher chose their pitch deterministically instead of randomly, the batter would have no uncertainty about the incoming pitch, e.g., if a pitcher only throws their fastball and never their curveball, batters know to expect a fastball. Denote the pitcher’s strategy set as ΔP, which is the set of all probability distributions over *P*. Thus, for p∈ΔP and $\pi =(\pi_{o},\pi_{u})\in P$, p(π) is the probability that the pitcher intends to throw pitch π with observable properties $\pi_{o}$ and unobservable properties $\pi_{u}$ under strategy *p*.

The batter’s action set is *S* = {swing, take}. The batter’s behavioral strategy is a probability distribution over *S* for each *information set*. In this context, an information set is a set of pitches that are indistinguishable to the batter given what they can observe about the pitch. For example, if we assume the batter can observe everything about the pitch before deciding to swing, then the batter has |*P*| information sets, one for each pitch in *P*. If we assume the batter can observe nothing about the pitch before deciding to swing, then the batter has just one information set corresponding to all of the pitches in *P* which are indistinguishable to the batter. The set of observable properties of the pitch $P_{o}$ define the information sets; the batter has one information set for every element of $P_{o}$ unless $P_{o}=\emptyset$, in which case the batter has only one information set. Denote ΔS as the set of probability distributions over *S* and [ΔS] as the set of possible behavioral strategies of the batter. For s∈[ΔS], denote $s_{\pi_{o}}\in \Delta S$ as the probability distribution the batter uses to sample an action under strategy *s* when they observe a pitch with observable properties $\pi_{o}\in P_{o}$. Thus for τ∈S, $s_{\pi_{o}}(\tau )$ is the probability that the batter takes action τ under strategy *s* after observing $\pi_{o}\in P_{o}$. This notation illustrates that the batter strategizes and takes action based on what they observe about the pitch (Fig 13).

Note that a behavioral strategy by definition is a probability distribution over actions in each of an agent’s information sets. We can define the pitcher’s behavioral strategy as a probability distribution over their action set *P* because the pitcher only has one information set since they make the first move in the game. Therefore, defining their strategy as a distribution over *P* is equivalent to defining their strategy as a distribution over *P* for each of their information sets.

Denote *C* as the set of all game contexts, where a game context is characterized by a batter, pitcher, and ball and strike count. Both the pitcher and batter have access to a utility function U:ΔP×[ΔS]×C→ℝ which outputs the expected run value given the pitcher strategy, batter strategy, and game context. Naturally, negative run values favor the pitcher, whereas positive run values favor the batter. Therefore, the pitcher’s goal is to use the pitching strategy p∈ΔP that minimizes *U*, and the batter’s goal is to use the strategy s∈[ΔS] that maximizes *U*. A pitching equilibrium is a pair of strategies $\left(p^{*},s^{*}\right)$ satisfying


$$\begin{array}{c} p^{*}\in \underset{p\in \Delta P}{\text{arg min}}\max_{s\in [\Delta S]}U(p,s,c) \end{array}$$ (4a)

$$\begin{array}{c} s^{*}\in \underset{s\in [\Delta S]}{\text{arg max}}\min_{p\in \Delta P}U(p,s,c). \end{array}$$ (4b)

Since the utility function *U* is the expected run value given a pitcher strategy, batter strategy, and game context, it is defined by first defining the expected run value of a pitch $\left(\pi_{o},\pi_{u}\right)$, batter swing decision τ, and game context *c*


$$\begin{array}{c} r(\pi_{o},\pi_{u},\tau ,c)=\sum_{x\in 𝒳}𝒫(x|\pi_{o},\pi_{u},\tau ,c)v(x,c), \end{array}$$ (5)

where 𝒳 is the set of possible pitch outcomes, 𝒳= {called ball (B), called strike (CS), swinging strike (SS), foul ball (F), ball in play out (O), single (1B), double (2B), triple (3B), home run (HR)}, and *v*(*x*,*c*) is the run value of a pitch outcome *x* in a specific game context *c*, where the relevant game context is simply the ball and strike count. The values of *v*(*x*,*c*) that were used were adapted from [19]. The conditional probabilities $𝒫(x|\pi_{o},\pi_{u},\tau ,c)$ are estimated using a batter-specific pitch outcome prediction model called OptimusPitch. More details about OptimusPitch are provided in Appendix B.

Recall the pitcher does not always hit their intended target, so for every intended pitch $(\pi_{o},\pi_{u})\in P$ we define a distribution over actual pitches $({\tilde{\pi }}_{o},{\tilde{\pi }}_{u})\in P$ as $𝒫({\tilde{\pi }}_{o},{\tilde{\pi }}_{u}|\pi_{o},\pi_{u})$. This distribution, which is termed the pitcher’s *execution error*, is estimated using a skill-estimation method called JEEDS, which is described in Appendix C. The pitcher should consider their execution error when strategizing, so the next step in defining the utility function is to take the expected value of (5) over this execution error distribution. Additionally, the batter’s behavioral strategy defines a distribution over *S* for the observed part of the actual pitch, $s_{{\tilde{\pi }}_{o}}$, so we take the expected value over that distribution as well. Finally, we take the expected value over the pitcher’s behavioral strategy *p*, so the utility function is ultimately defined as


$$\begin{array}{c} U(p,s,c)=\sum_{\begin{array}{c} \pi_{o}\in P_{o}\\ \pi_{u}\in P_{u} \end{array}}p((\pi_{o},\pi_{u}))\sum_{\tau \in S}\sum_{\begin{array}{c} {\tilde{\pi }}_{o}\in P_{o}\\ {\tilde{\pi }}_{u}\in P_{u} \end{array}}s_{{\tilde{\pi }}_{o}}(\tau )𝒫({\tilde{\pi }}_{o},{\tilde{\pi }}_{u}|\pi_{o},\pi_{u})r({\tilde{\pi }}_{o},{\tilde{\pi }}_{u},\tau ,c). \end{array}$$ (6)

The first summation in (6) gives the expected value over the pitcher’s strategy, the second summation gives the expected value over the batter’s strategy, and the third summation gives the expected value over the pitcher’s execution error distributions.

It takes some small amount of time for a batter to complete their swing. Naturally, there is a decision point along a pitch’s trajectory where if the batter has not decided to swing by that point, they no longer have enough time to complete their swing before the ball reaches the catcher’s glove. Therefore, a realistic assumption is that the batter can only observe the full pitch up to this decision point. A pitcher can throw multiple pitch types through the same decision point, so the batter does not know exactly where the pitch will end up after the decision point because they are not sure which pitch type the pitcher threw. For example, a fastball that ends up at the top of the strike zone may share a decision point with a curveball that ends up at the bottom of the strike zone (Fig 12). Pitchers often use a pitching strategy known as tunneling where they try to throw consecutive pitches of different types along a similar trajectory up to the decision point [20]. We will therefore use the terms “decision point” and “tunnel point” interchangeably throughout the remainder of this paper.

**Fig 12:**
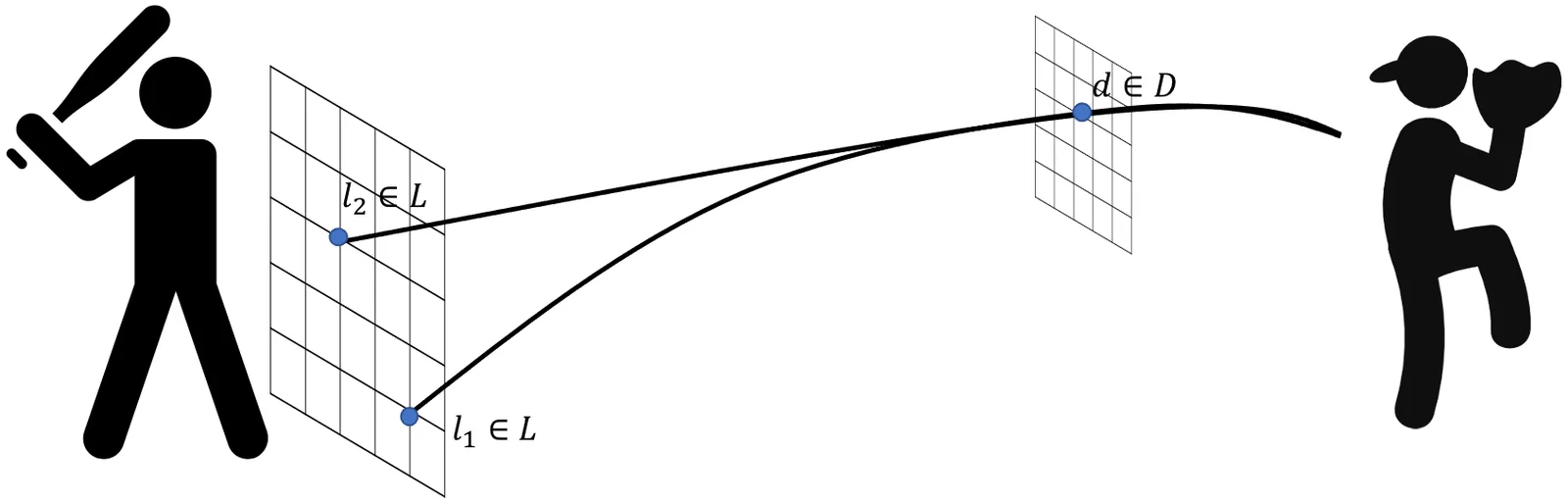
Visualizing the decision points. A comparison of the sets *D* and *L*. The set *L* is the set of location points in the strike zone. The set *D*, on the other hand, is a set of decision points, which represent where the ball is when the batter needs to make a decision whether to swing. Note that a single point in *D* could correspond to several points in *L*, depending on the pitch type. One way to model the interaction is to say $P_{o}$ is every property about the pitch that can be observed before *d* and $P_{u}$ is everything that happens after *d* [18].

Denote *D* as the grid of possible decision points and *T* as the set of possible pitch types. We assume that the batter can observe the decision point but not the pitch type, so we have $P_{o}=D$ and $P_{u}=T$. The decision point was set at 24 feet from home plate based on [21]. Denote *L* as the grid of possible pitch locations at home plate. [18] defined a mapping from D×T to *L* using the work in [22–24]. This was required for the utility function since the model of $𝒫(x|\pi_{o},\pi_{u},\tau ,c)$ in (5) depends on the pitch’s location at home plate rather than its location at the decision point.

Note, in [18], they acknowledge that the decision point model is unrealistic for human batters. Some batters may be better or worse at processing information that they can observe about the pitch, and they all have slightly different reaction times. Additionally, if the resulting strategy is a behavioral strategy, then the batter needs some way to randomize their decision to swing, which is impossible. However, that is not a problem for the resulting pitching equilibrium strategy. By definition, if the batter deviates from their equilibrium swinging strategy because it is impossible for them to actually implement that strategy, the pitcher can only gain from that. Thus, a pitching strategy that is optimal against a hypothetical batter who is impossibly good at processing information about a pitch will perform even better against a real human batter who cannot process information as well.

Substituting *D* for $P_{o}$ and *T* for $P_{u}$, the utility function (6) becomes


$$\begin{array}{cc} U(p,s,c) & =\sum_{\begin{array}{c} d\in D\\ t\in T \end{array}}p((d,t))\sum_{\tau \in S}\sum_{\begin{array}{c} \tilde{d}\in D\\ \tilde{t}\in T \end{array}}s_{\tilde{d}}(\tau )𝒫(\tilde{d},\tilde{t}|d,t)r(\tilde{d},\tilde{t},\tau ,c)\\ & =\sum_{\begin{array}{c} d\in D\\ t\in T \end{array}}p((d,t))\sum_{\tau \in S}\sum_{\tilde{d}\in D}s_{\tilde{d}}(\tau )𝒫(\tilde{d}|d,t)r(\tilde{d},t,\tau ,c), \end{array}$$ (7)

where the second equality follows because the execution error distribution $𝒫(\tilde{d},\tilde{t}|d,t)$ is a distribution over pitch locations/decision points given a pitch type. We assume that the pitcher will always throw their intended pitch type without any execution error, meaning if they intend to throw a 93 mph fastball, then they throw a 93 mph fastball. A more realistic assumption is that when they intend to throw a 93 mph fastball, the actual fastball they throw is sampled from a distribution of fastballs with a range of possible velocities and trajectories. However, the assumption that the pitcher always throws their intended pitch type simplifies computation by implying that


$$\begin{array}{c} 𝒫(\tilde{d},\tilde{t}|d,t)=\left\{ \begin{array}{cc} 0 & \text{ if }t\text{≠}\tilde{t}\\ 𝒫(\tilde{d}|d,t) & \text{ if }t=\tilde{t}. \end{array}\right. \end{array}$$

This fact allows us to remove the sum over $\tilde{t}\in T$ from the utility function since $\tilde{t}\text{≠}t$ implies $𝒫(\tilde{d},\tilde{t}|d,t)=0$. Of course, the assumption could be removed if a baseball team wanted to find a slightly more realistic equilibrium.

Let *A* be a matrix who’s (*i*, *j*)^th^ entry is given by


$$\begin{array}{c} 𝒫({\tilde{d}}_{j}|d_{i},t_{i})r({\tilde{d}}_{j},t_{i},\tau_{j},c) \end{array}$$

where $\left(d_{i},t_{i}\right)$ is the $i^{th}$ pitch in D×T, ${\tilde{d}}_{j}$ is the $\frac{j}{2}$ tunnel in *D* if *j* is even and the $\frac{j+1}{2}$ tunnel in *D* if *j* is odd, and $\tau_{j}\in S$ is the action of taking a pitch in tunnel ${\tilde{d}}_{j}$ if *j* is even and swinging at a pitch in tunnel ${\tilde{d}}_{j}$ if *j* is odd. For instance, $\tau_{1}$ corresponds to swinging at a pitch in tunnel 1 and $\tau_{2}$ corresponds to taking a pitch in tunnel 1. Thus, *A* is |D||T|×2|D| because there is a row for every choice of $(d_{i},t_{i})\in D\times T$ and two columns for every tunnel in *D*, one corresponding to swinging at a pitch in that tunnel, and one corresponding to taking a pitch in that tunnel. We can arrange the batter behavioral strategy s∈[ΔS] into a vector 𝐬 of length 2|*D*| where the *j*^th^ entry is the probability of taking action $\tau_{j}$ under strategy *s*. In other words, it is the probability of swinging at a pitch in tunnel $\frac{j+1}{2}$ if *j* is odd and taking a pitch in tunnel $\frac{j}{2}$ if *j* is even. Then right multiplying *A* with this strategy vector 𝐬 is equivalent to taking the weighted sum of $𝒫({\tilde{d}}_{j}|d_{i},t_{i})r({\tilde{d}}_{j},t_{i},\tau_{j},c)$ over all τ∈S and all tunnels ${\tilde{d}}_{j}\in D$, where the weights are given by the batter’s strategy for that tunnel $s_{{\tilde{d}}_{j}}(\tau_{j})$. In other words, the $i^{th}$ entry of the vector A𝐬 is defined as


$$\left[A𝐬{]}_{i}=\sum_{\tau \in S}\sum_{\tilde{d}\in D}s_{\tilde{d}}(\tau )𝒫(\tilde{d}|d_{i},t_{i})r(\tilde{d},t_{i},\tau ,c\right)$$

which is the expected run value when the pitcher chooses intended pitch $(d_{i},t_{i})\in P$ against a batter who is playing strategy *s*. We can therefore get the expected value over the pitcher’s strategy p∈ΔP by left multiplying A𝐬 with the row vector version of the pitcher’s strategy, ${𝐩}^{T}$. That gives


$$\begin{array}{c} {𝐩}^{T}A𝐬=\sum_{\begin{array}{c} d\in D\\ t\in T \end{array}}p((d,t))\sum_{\tau \in S}\sum_{\tilde{d}\in D}s_{\tilde{d}}(\tau )𝒫(\tilde{d}|d,t)r(\tilde{d},t,\tau ,c), \end{array}$$ (8)

which is of course equivalent to the utility function (7). Thus, the equilibrium in (4) can be redefined as a pair of vectors $\left({𝐩}^{*},{𝐬}^{*}\right)$ that satisfy


$$\begin{array}{c} {𝐩}^{*}\in \underset{𝐩}{\text{arg min}}\max_{𝐬}{𝐩}^{T}A𝐬 \end{array}$$ (9a)

$$\begin{array}{c} {𝐬}^{*}\in \underset{𝐬}{\text{arg max}}\min_{𝐩}{𝐩}^{T}A𝐬. \end{array}$$ (9b)

This formulation of the utility function results in a linear programming problem whose solution is an equilibrium:


$$\begin{array}{c} \begin{array}{cc} \max_{𝐬,v}\; & v\\ \text{subject to }\; & A𝐬⪰v\\ & s_{i}+s_{i+1}=1\text{ for every }i\in D\\ & 𝐬⪰0. \end{array} \end{array}$$

The constraints ensure that 𝐬 represents a behavioral strategy because the values of 𝐬 in each tunnel are nonnegative and sum to 1. The solution to this problem gives the equilibrium strategy for the batter, ${𝐬}^{*}$, as well as the equilibrium utility $v^{*}={𝐩}^{*T}A{𝐬}^{*}$. Solving the dual of this problem gives the pitcher’s equilibrium strategy ${𝐩}^{*}$. ${𝐩}^{*}$ defines an effective pitching strategy that is robust to batter counterstrategies. However, ${𝐩}^{*}$ does not consider the effect that the defense’s positioning strategy could have on the batter’s strategy nor how that changes the optimal pitching strategy. We address that issue in the next subsection by incorporating the positioning strategy into the equilibrium.

To conclude this subsection, we depict an example of the pitching game in extensive form (Fig 13). In this example, suppose $|T|=n_{t}$ and $|D|=n_{d}$. The pitcher plays first in the game by randomly sampling an intended pitch $\left(d_{i},t_{j}\right)$ from D×T according to their behavioral strategy *p*. The choice of intended pitch results in a chance node where the actual decision point $\tilde{d}$ is randomly chosen according to the execution error probability distribution $𝒫(\tilde{d}|d_{i},t_{j})$. The batter can observe the actual decision point $\tilde{d}$, but they cannot observe the pitcher’s choice of pitch type, so after the chance node we move into one of $n_{d}$ information sets defined by $\tilde{d}$ and depicted in Fig 13 by the dashed oval. There are $n_{t}$ choice nodes in each information set because the batter does not know which pitch type in *T* that the pitcher chose to throw through $\tilde{d}$. The batter plays the same strategy, $s_{\tilde{d}}$ for every choice node in the information set because they can not distinguish between those nodes. Thus, although the batter’s strategy *s* is defined by a distribution over actions in each possible information set, on any individual pitch the only relevant part of their strategy is the strategy they use in the observed information set $s_{\tilde{d}}$. The payoff is defined after the batter takes their action τ by $r(\tilde{d},t,\tau ,c)$, but the overall game utility *U*(*p*, *s*, *c*) is the expected value of *r* over the batter’s strategy, execution error distribution, and the pitcher’s strategy.

**Fig 13:**
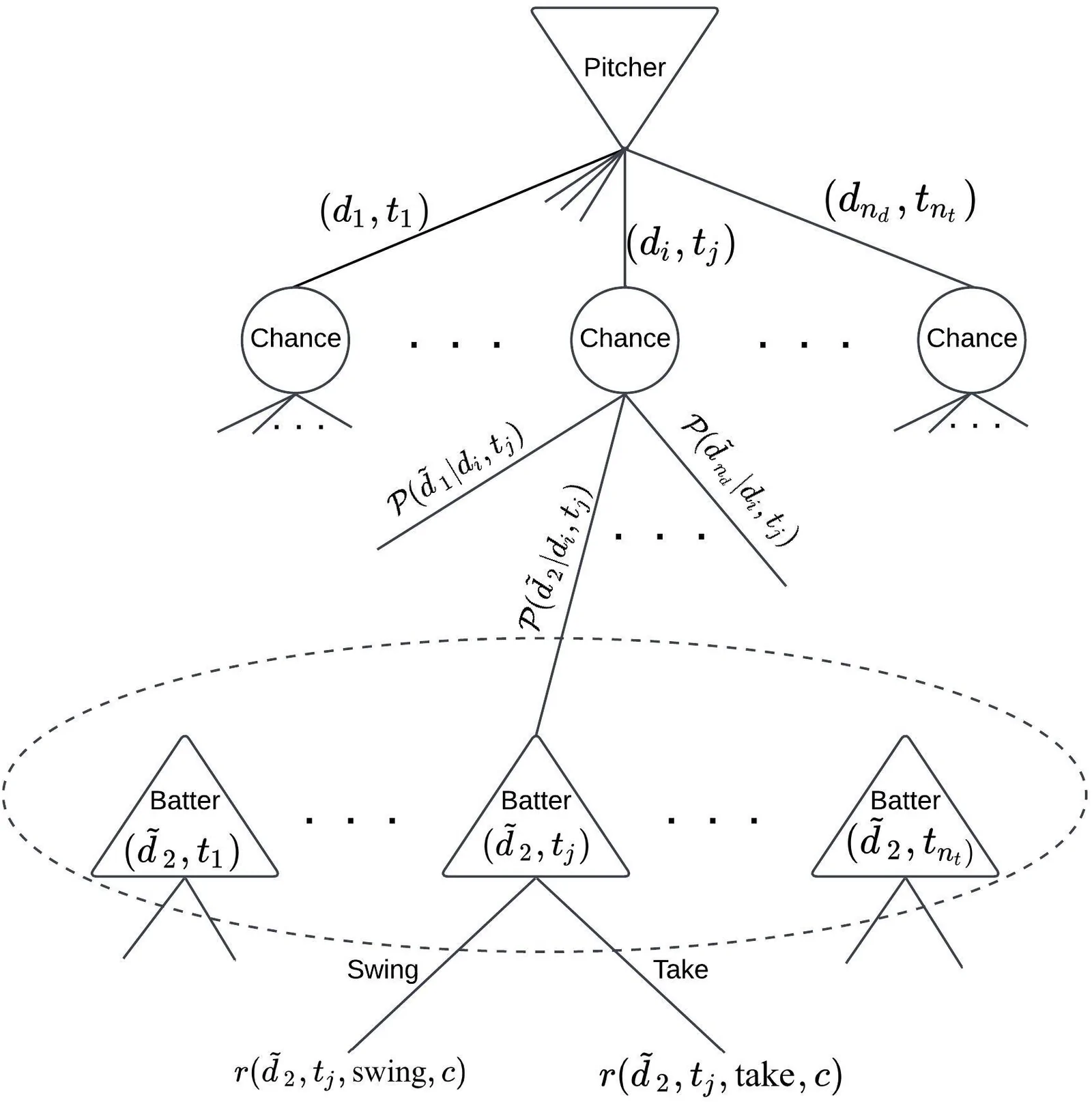
The pitching game in extensive form. The pitcher plays first by choosing an intended pitch $\left(d_{i},t_{j}\right)$. The choice of intended pitch determines the execution error distribution, which is modeled in the extensive form as a chance player that chooses an actual $\tilde{d}$ according to $𝒫(\tilde{d}|d_{i},t_{j})$. The chance player’s choice of $\tilde{d}$ defines the information set, which is depicted using the dashed oval. There is one node for each pitch type *t* in each information set because the batter can observe $\tilde{d}$ but not *t*. The batter’s strategy *s* is a distribution over actions for each informa*t*ion set, but for an actual pitch the only relevant part of their strategy is the strategy they use in the information set they observe, $s_{\tilde{d}}$. The terminal value in the game is defined after the batter chooses their action τ as $r(\tilde{d},t,\tau ,c)$, and the overall game utility is the expected value of *r* over the batter’s strategy, the execution error distribution, and the pitcher’s strategy. In this figure, we followed the path of the tree where the pitcher chose intended pitch $\left(d_{i},t_{j}\right)$ and the actual pitch determined by the chance node was $\left({\tilde{d}}_{2},t_{j}\right)$, so the batter would have chosen their action according to $s_{{\tilde{d}}_{2}}$.

#### 2.3.2. Incorporating positioning into the pitching equilibrium.

In this subsection we describe our contribution to the pitching game, which is the addition of a complementary positioning strategy resulting in a complete defensive strategy. We begin by defining an alternative expected run value function (5) that considers the positioning strategy, ψ. Partition the pitch outcome set 𝒳 into the set of ball in play (BIP) outcomes, ${𝒳}_{\text{BIP}}=$ {O, 1B, 2B, 3B, HR}, and non-ball in play outcomes ${𝒳}_{\neg \text{BIP}}=$ {B, CS, SS, FB}. We can effectively predict the probability of a ball in play given a pitch, swing decision, and game context by adding up the probabilities of the ball in play outcomes,


$$\begin{array}{c} 𝒫(\text{BIP}|\pi_{o},\pi_{u},\tau ,c)=\sum_{x\in {𝒳}_{\text{BIP}}}𝒫(x|\pi_{o},\pi_{u},\tau ,c). \end{array}$$

Denote ${𝐛}_{\pi }=[b_{1},b_{2},...,b_{N}]$ as a sample of *N* batted balls from the pitch-specific batted ball trajectory distribution for pitch π∈P. We define the expected run value of a BIP given the pitch π and a positioning strategy ψ as


$$\begin{array}{c} r_{\text{BIP}}(\pi ,\psi ,c)=\frac{1}{N}\sum_{j=1}^{N}g_{\text{runs}}(b_{j},\psi ,c), \end{array}$$

where *g*_runs_ is the same function as the wOBA objective function *f*_woba_ only we use the context specific run values from [19] instead of the wOBA weights, hence why *g*_runs_ is a function of the game context *c* and *f*_woba_ is not. We then define the alternative to *r* in (5) that considers the positioning strategy ψ as


$$\begin{array}{c} \phi (\pi_{o},\pi_{u},\tau ,c,\psi )=\sum_{x\in {𝒳}_{\neg \text{BIP}}}[𝒫(x|\pi_{o},\pi_{u},\tau ,c)v(x,c)]+𝒫(\text{BIP}|\pi_{o},\pi_{u},\tau ,c)r_{\text{BIP}}(\pi_{o},\pi_{u},\psi ,c). \end{array}$$ (10)

We define a positioning specific utility function by replacing $r(\pi_{o},\pi_{u},\tau ,c)$ in (6), (7), and (8) with $\phi (\pi_{o},\pi_{u},\tau ,c,\psi )$.

Instead of choosing a pitching strategy to minimize utility, the defense now has to choose both a pitching strategy and a positioning strategy. Therefore, the complete defensive equilibrium is a positioning strategy $\psi^{*}$, pitching strategy $p^{*}$, and swing strategy $s^{*}$ satisfying


$$\begin{array}{cc} \psi^{*},p^{*}\in & \underset{\begin{array}{c} p\in \Delta P\\ \psi \in \Psi \end{array}}{\text{arg min}}\max_{s\in [\Delta S]}U(p,s,c,\psi )\\ s^{*}\in & \underset{s\in [\Delta S]}{\text{arg max}}\min_{\begin{array}{c} p\in \Delta P\\ \psi \in \Psi \end{array}}U(p,s,c,\psi ). \end{array}$$

This of course is the equilibrium we defined in (2). Since *A* is defined using ϕ, which is a function of the positioning strategy ψ, denote A(ψ) as the *A* matrix calculated under the positioning strategy ψ. We can then rewrite the matrix version of the pitching equilibrium (9) to include the positioning strategy as


$$\begin{array}{c} \psi^{*},{𝐩}^{*}\in \underset{\begin{array}{c} 𝐩\\ \psi \in \Psi \end{array}}{\text{arg min}}\max_{𝐬}{𝐩}^{T}A(\psi )𝐬 \end{array}$$ (11a)

$$\begin{array}{c} {𝐬}^{*}\in \underset{𝐬}{\text{arg max}}\min_{\begin{array}{c} 𝐩\\ \psi \in \Psi \end{array}}{𝐩}^{T}A(\psi )𝐬. \end{array}$$ (11b)

To simplify the computation of this equilibrium, we restrict the set of admissable positioning strategies Ψ to be a finite set of precomputed positioning strategies. For example, when we calculate this equilibrium for Nick Anderson versus Mookie Betts in the following subsection, we assume that the defense chooses between three different positioning strategies: two pitch-specific positioning strategies corresponding to Anderson’s fastball and curveball and one matchup-specific positioning strategy. That gives us a finite number of possible A(ψ) matrices. For each of those matrices, we calculate the pitching equilibrium (9). We then define the complete defensive equilibrium $\left(\psi^{*},{𝐩}^{*},{𝐬}^{*}\right)$ as the positioning strategy $\psi^{*}$ that resulted in the pitching equilibrium with minimum utility along with the corresponding batter and pitcher strategies ${𝐬}^{*}$ and $p^{*}$. We believe the restriction of Ψ to a finite number of positioning strategies is justifiable because fielders would likely not be able to execute more than a small finite number of positioning strategies per batter.

Note, some batters may try to change their batted ball trajectories in response to the defense’s positioning strategy. For example, a hitter may try to go more oppo if the defense is playing them to pull the ball or a left-handed hitter may try to bunt down the third baseline if the third baseman is playing too deep. We can strategize for adaptable hitters like these by expanding the batter action set *S*. Instead of having *S* = {swing, take}, we would have *S* = {swing, bunt, take} or *S* = {swing pull, swing oppo, take}. In fact, we can expand *S* to include any finite number of possible batted ball trajectory distributions for the batter, and we solve for the complete defensive strategy using the exact same method as in (11). The only difference is that the *A* matrix has |*S*||*D*| columns instead of 2|*D*| columns, one column for each action in *S* and each decision point in *D*.

#### 2.3.3. Demonstration: Anderson versus Betts.

On October 27, 2020, the Tampa Bay Rays faced off against the Los Angeles Dodgers in Game Six of the World Series. This game is most remembered for Rays manager Kevin Cash’s controversial decision to relieve starting pitcher Blake Snell in the sixth inning, despite a dominant performance from Snell who had held the Dodgers scoreless. Unfortunately for the Rays, this decision immediately backfired as the relief pitcher, Nick Anderson, gave up a double to the first batter he faced, Mookie Betts. One wild pitch and RBI later and the Dodgers took the lead, ultimately winning both the game and the series. In this section, we illustrate the complete defensive strategy in this matchup between Nick Anderson and Mookie Betts.

Nick Anderson throws two different pitch types: a fastball and a curveball. Thus, *T* = {fastball, curveball}. We defined *D* as the grid of baseball sized (3 inch) squares 24 feet from home plate that range from −2.5 to 2.5 feet in the horizontal direction and 2–7 feet in the vertical direction (where the origin is the back tip of home plate). Recall, when we solve for the complete defensive strategy in (11), we precompute a finite set of admissible positioning strategies Ψ. Fig 14 shows these strategies: a matchup-specific (m), fastball-specific (ff) and curveball-specific (cu) strategy. Thus, Ψ= {m, ff, cu}. Note, there was a runner on first base when Anderson faced Betts, so the first baseman’s position was fixed at first base for all three positioning strategies to hold the baserunner.

**Fig 14:**
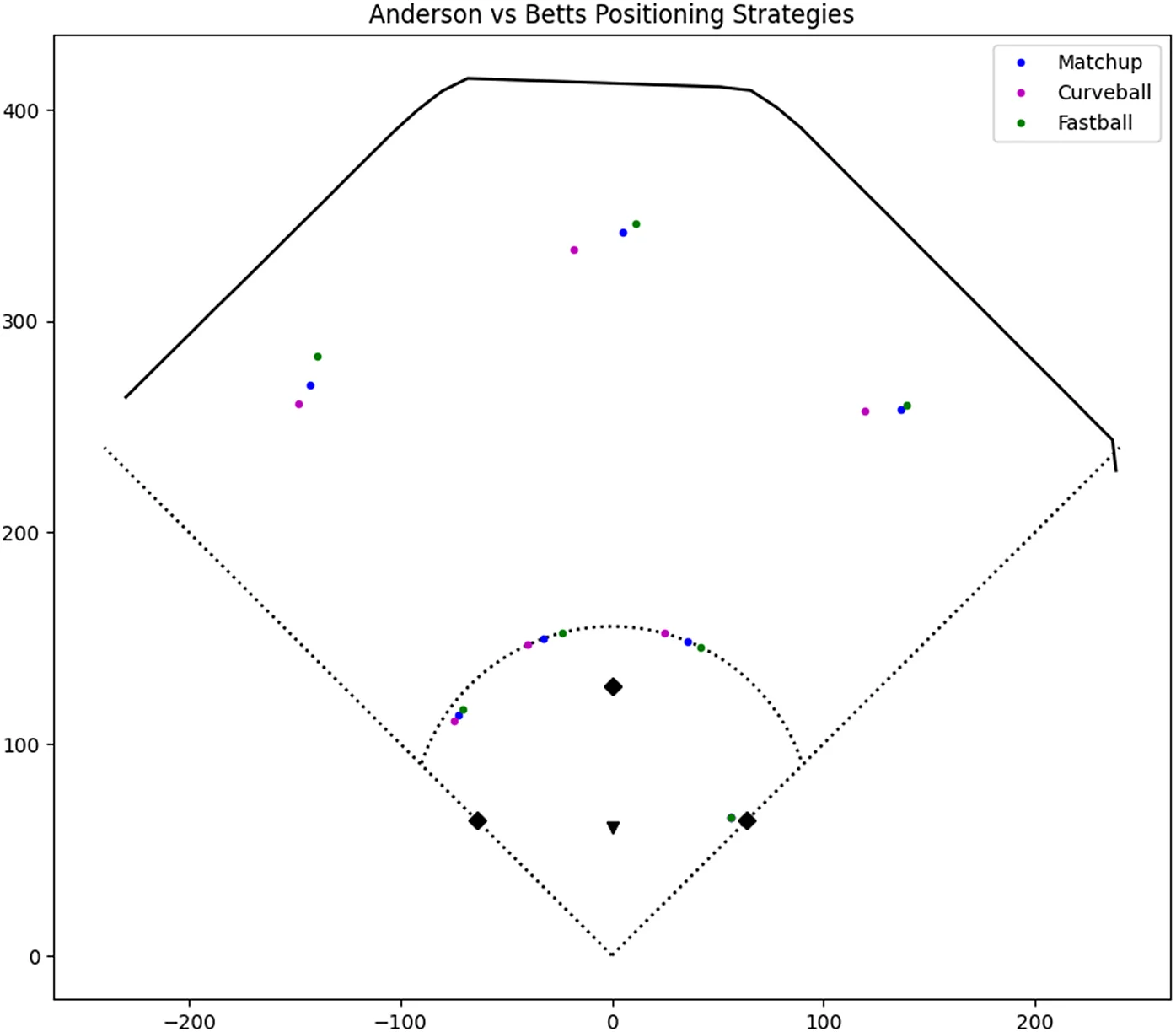
The matchup-specific, fastball-specific, and curveball-specific positioning strategies for Nick Anderson versus Mookie Betts.

A summary of the complete defensive strategy for each ball and strike count is shown in Table 2. The first column gives the recommended positioning strategy, the second column gives the rate that Anderson should throw his fastball, the third column gives the rate that he should throw his curveball, and the fourth gives the equilibrium utility. The defense only uses the curveball or matchup positioning strategies. When they use the curveball positioning strategy, the optimal pitching strategy tends to favor the curveball over the fastball. Likewise, when they use the matchup positioning strategy, the optimal pitching strategy favors the fastball over the curveball. However, Anderson does not let the positioning strategy tip the pitch to Betts because he mixes between pitches in each positioning strategy. In other words, he mixes in an occasional bluff so that Betts cannot use the positioning strategy to perfectly predict the pitch. Notably, in the 3−0 count strategy, Anderson actually bluffs more frequently than not by throwing his fastball a majority of the time even though the defense is using the curveball positioning strategy. Fig 15 shows the pitching strategy for the 3−0 count.

**Table 2:** The complete defensive strategy for Nick Anderson versus Mookie Betts.

Count	Pos. Strat.	FF Rate	CU Rate	Utility
0−0	m	0.63	0.37	0.011
0-1	m	0.66	0.34	0.008
0-2	m	0.64	0.36	0.006
1−0	m	0.65	0.35	0.011
1−1	m	0.62	0.38	0.012
1-2	m	0.64	0.36	0.014
2−0	cu	0.48	0.52	0.007
2−1	m	0.60	0.40	0.016
2−2	m	0.63	0.37	0.018
3−0	cu	0.52	0.48	−0.006
3−1	cu	0.48	0.52	0.025
3−2	cu	0.49	0.51	0.02

**Fig 15:**
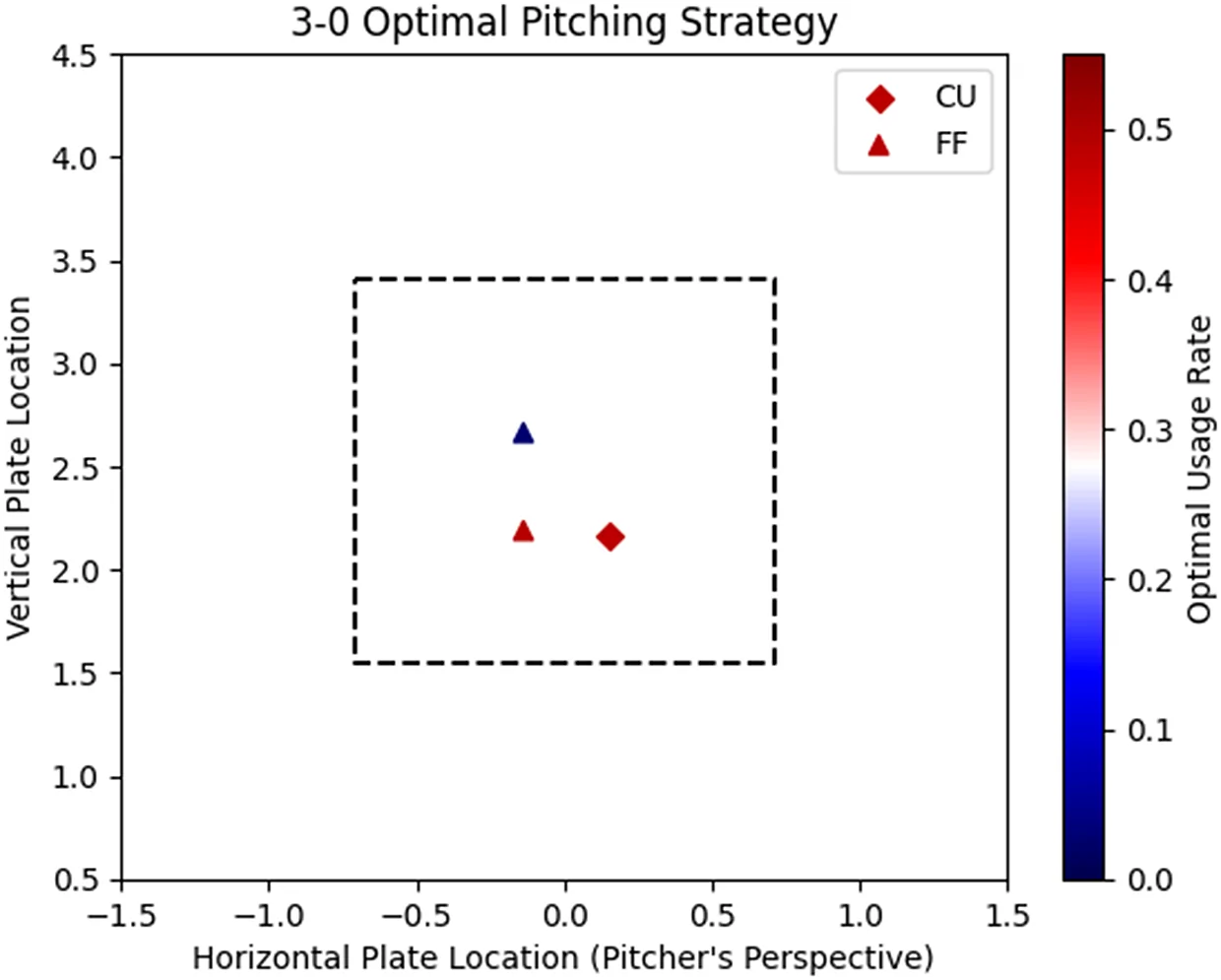
The 3-0 pitching strategy. The optimal pitching strategy in the 3-0 count where Anderson bluffs more frequently than not by throwing his fastball slightly more frequently than his curveball even though the defense is positioned in the curveball positioning strategy.

Betts hit his double against Anderson in the third pitch of the at bat in a 2-0 count. Notably, the complete defensive strategy recommends that the defense switches from the matchup positioning to the curveball positioning when the count goes from 1-0 to 2-0. Had the Rays been following the complete defensive strategy, all the fielders would have noticeably shifted more towards the pull side. From a practical perspective, this signals to Betts that he should expect to see a curveball with higher likelihood because he is more likely to pull a curveball than a fastball. This phenomenon is modeled in our game theory model by the choice of *A* matrix. The batter gets to observe the defense’s choice of *A* matrix because they can observe the defense’s positioning. If some pitches are preferable to the pitcher in certain positioning strategies, that will be reflected by the payoffs in the *A* matrix. Thus, in the same way that Betts knows that Anderson prefers to throw more curveballs when the defense shifts towards the pull side, a batter in the complete defensive strategy game knows which pitches a pitcher prefers to throw given the defense’s choice of *A* matrix. Fig 16 shows the optimal pitching strategy in the 2−0 count as well as the actual pitch Anderson threw that Betts hit for a double. The actual fastball was close to the aim point of the equilibrium fastball, so it looks like Anderson chose a good pitch even though the result was not ideal.

**Fig 16:**
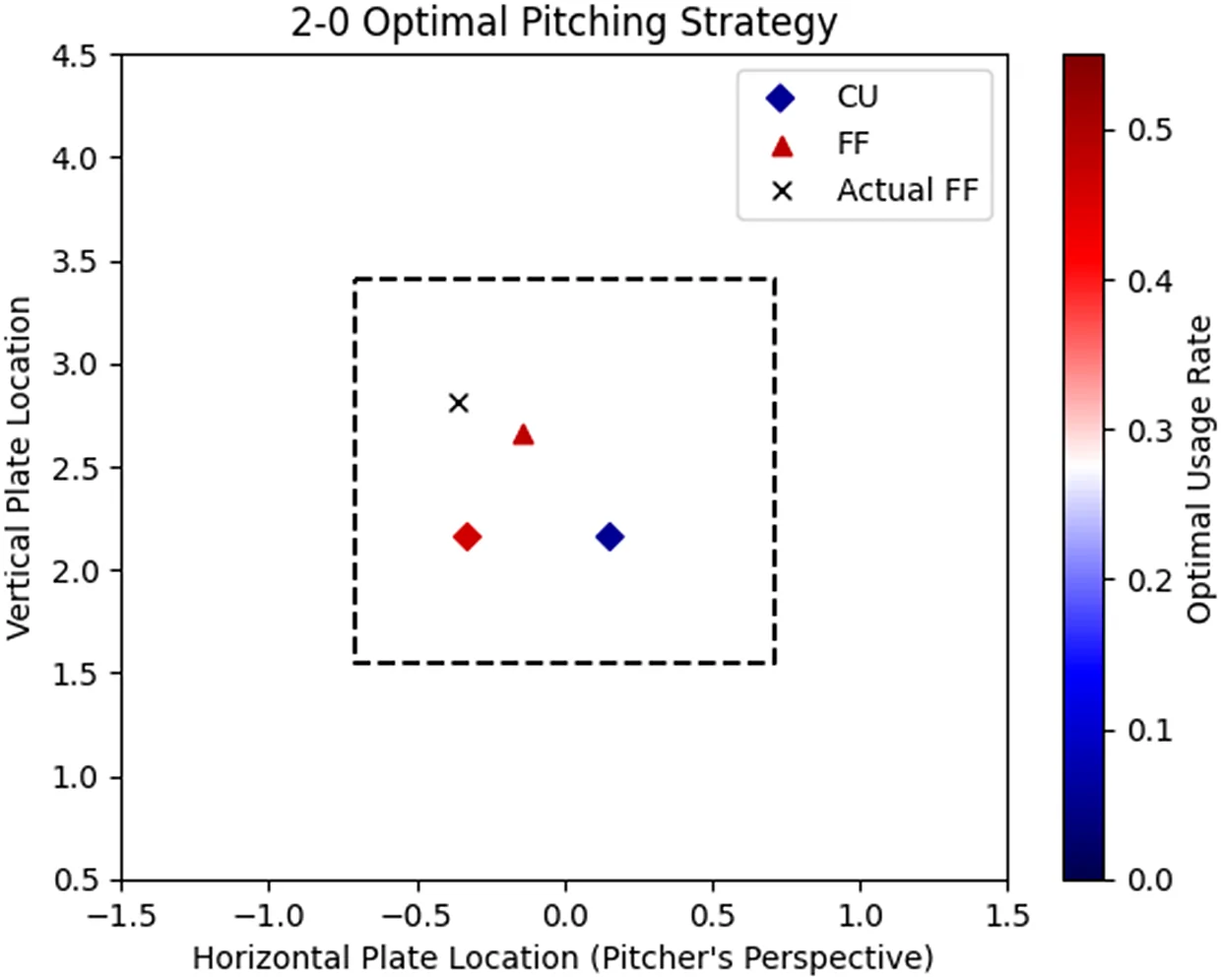
The 2-0 pitching strategy. The optimal pitching strategy in the 2−0 count where Betts hit the double. The actual pitch was a fastball that was close to the aim point of the fastball in the equilibrium strategy. Thus, it appears that Anderson threw a good pitch, but Betts just happened to make a better swing.

In practice, a team could define the complete defensive strategy for every one of their pitchers against every opposing batter before a game. They could give the fielders positioning cards telling them where to stand for each batter and pitcher and ball and strike count. Additionally, although it is very uncommon in MLB for anyone besides the pitcher or catcher to call the pitches, there have been some recent cases where teams had a pitching coach call pitches from the dugout [25]. Teams could call pitches from the dugout by using a random number book to sample a pitch from the optimal pitching strategy. Thus, if a team wanted to get the benefit of the pitch-specific positioning strategies without tipping the pitch to the opposing batter, they could plausibly do that with the complete defensive strategy. However, it is clearly significantly more complex to utilize the complete defensive strategy than it is to use a simple, batter-specific strategy. Additionally, the value added by using such a strategy is insignificant, as evidenced by Table 1. We suggest that most baseball teams are better off using a simple batter-specific positioning strategy rather than the complete defensive strategy.

#### 2.3.4. Comparing the complete defensive strategy and the pitch-specific positioning strategy.

By including the matchup-specific positioning strategy as one of the candidate positioning strategies for the complete defensive strategy, we guarantee that the complete defensive strategy will always be at least as effective as the matchup-specific positioning strategy. It seems obvious that the complete defensive strategy will also be better than the pitch-specific positioning strategy because it does not tip the pitch to the batter. However, it is possible that the value of the pitch-specific positioning outweighs the cost of tipping the pitch. In this section, we investigate that question.

Recall, in the pitching game we partition the pitch into the set of observable and unobservable properties $P_{o}$ and $P_{u}$. In the complete defensive strategy, we assume that $P_{o}=D$ and $P_{u}=T$. If the defense chose to use the pitch-specific positioning strategy for every pitch, then we would have $P_{o}=D\times T$ and $P_{u}=\emptyset$. Equivalently, we would have $P_{o}=L\times T$ and $P_{u}=\emptyset$ because there is a known mapping from D×T to *L*. In other words, if the defense uses the pitch-specific positioning strategy corresponding to the pitcher’s pitch, we assume the batter can observe the full trajectory of the pitch when making their swing decision.

In [18], they also define the optimal pitching strategy when $P_{o}=L\times T$. In a perfect information game, a pure strategy Nash equilibrium exists, which is an equilibrium that does not require randomizing. Thus, the optimal pitching strategy can be defined as the single intended pitch that minimizes expected utility under the assumption that the batter will observe the full pitch trajectory and make the maximizing swing decision for the pitch they observe. Table 3 shows the optimal pitch type and the equilibrium utility in the pitching game when the defense chooses to tip the pitch by using the pitch-specific positioning strategy that corresponds to the choice of pitch type. It also shows the equilibrium utilities from the complete defensive strategy (Table 2). Not surprisingly, the pitch-specific approach to positioning has a worse equilibrium utility in every ball and strike count than the complete defensive strategy. The value of using the pitch-specific positioning strategy does not outweigh the cost of tipping the pitch.

**Table 3:** Comparison of a pitch-tipping positioning strategy with the complete defensive strategy.

Count	Pitch Type	Pitch Tipping Utility	C.D.S. Utility
0−0	Fastball	0.016	0.011
0-1	Fastball	0.013	0.008
0-2	Fastball	0.019	0.006
1−0	Fastball	0.017	0.011
1−1	Fastball	0.019	0.012
1-2	Fastball	0.033	0.014
2−0	Fastball	0.015	0.007
2−1	Fastball	0.026	0.016
2−2	Fastball	0.044	0.018
3−0	Fastball	−0.001	−0.006
3−1	Curveball	0.036	0.025
3−2	Fastball	0.064	0.02

## 3. Conclusion

In this paper, we addressed the common criticism that positioning strategies do not complement the pitching strategy. We learned novel pitch-specific batted ball trajectory distributions and showed how they can be used to define matchup and pitch-specific positioning strategies. To implement the pitch-specific positioning strategy without tipping the pitch to the batter, we combined our pitch-specific positioning strategies with an existing pitching model to determine how frequently a pitcher needs to bluff in order for the value of the pitch-specific positioning to outweigh the cost of revealing more information about the upcoming pitch. We call the complementary positioning and pitching strategies the complete defensive strategy. The complete defensive strategy allows baseball defenses to utilize the most effective pitch-specific positioning strategies without revealing too much information about the upcoming pitch to the batter. In an illustrative example where pitcher Nick Anderson faced off against batter Mookie Betts, we found that the complete defensive strategy was more effective than both the matchup-specific positioning strategy that does not risk tipping the pitch to the batter and the pitch-specific positioning strategy that completely reveals the pitch to the opposing batter. Ultimately, we found that positioning strategies that incorporate information about the pitch/pitcher are not significantly more effective than strategies that only consider the batters’ tendencies, but they are significantly more difficult to implement. We therefore conclude that the criticism that positioning strategies do not complement the pitching strategy is unwarranted, and most baseball teams would likely be better off using a simple batter-specific positioning strategy rather than the complete defensive strategy.

## Appendix

### A. The objective functions

Both the outs objective function *f*_outs_ and the wOBA objective function *f*_woba_ depend on the probability of an out given a batted ball trajectory and a positioning strategy. [7] modeled the out probability using two separate logistic regression models: one for the probability of an out on a ground ball and the other for the probability of an out on a non-ground ball. The ground ball out probability model is given by


$$\begin{array}{c} f_{\text{gb}}(b,\psi )=\sigma (-2.78-0.15a_{d}+5.87b_{t}-1.32b_{t}^{2}) \end{array}$$ (10)

where *b* is the batted ball’s trajectory, ψ is the defense’s positioning strategy, $a_{d}$ is the minimum absolute difference between the spray angle of the batted ball and the spray angle of the infielders’ starting positions, $b_{t}$ is the “ball time to fielder” which is the depth from home plate of the closest fielder divided by the exit speed of the ground ball, and σ is the sigmoid function, $\sigma (x)=1/(1+e^{-x})$. It is appropriate to define *f*_gb_ as a function of *b* and ψ because $a_{d}$ and $b_{t}$ depend on both the batted ball’s trajectory and the defense’s positioning strategy.

[7] defined the non-ground ball out probability model as


$$\begin{array}{c} f_{\neg \text{gb}}(b,\psi )=\sigma (-2.17+1.5\delta_{t}) \end{array}$$ (11)

where σ is the sigmoid function, *b* is the batted ball’s trajectory, ψ is the positioning strategy, and $\delta_{t}$ is the difference between a batted ball’s hang time and the time that it takes the closest fielder to reach the landing spot of the batted ball if they are assumed to be moving at 27 feet per second (MLB average sprint speed). In other words, $\delta_{t}=\text{hang time}-(\text{distance to ball}/27)$, where hang time is measured in seconds and distance in feet. It is therefore appropriate to define $f_{\neg \text{gb}}$ as a function of *b* and ψ because $\delta_{t}$ depends on the batted ball’s trajectory and the fielders’ positions.

Combining the ground ball (12) and non-ground ball (13) outs models [7], defined the probability of an out given a batted ball and positioning strategy as


$$\begin{array}{c} 𝒫(\text{out}|b,\psi )=\left\{ \begin{array}{c} f_{\text{gb}}(b,\psi )\text{ if }b\text{ is a ground ball}\\ f_{\neg \text{gb}}(b,\psi )\text{ if }b\text{ is not a ground ball}. \end{array}\right. \end{array}$$ (12)

Minimizing negative expected outs is equivalent to maximizing expected outs, so the outs objective function is


$$\begin{array}{c} f_{\text{outs}}(b,\psi )=-𝒫(\text{out}|b,\psi ). \end{array}$$ (13)

The wOBA objective function is


$$\begin{array}{c} f_{\text{woba}}(b,\psi )=(1-𝒫(\text{out}|b,\psi ))\cdot g(b), \end{array}$$ (14)

where 𝒫(out|b,ψ) is their outs model (14) and *g*(*b*) is the expected wOBA of a batted ball given that it is not an out. That is


$$\begin{array}{c} g(b)=w_{1}𝒫(1\text{B}|b)+w_{2}𝒫(2\text{B}|b)+w_{3}𝒫(3\text{B}|b)+w_{4}𝒫(\text{HR}|b) \end{array}$$

where *w*_1_, *w*_2_, *w*_3_, and *w*_4_ are the wOBA weights of a single, double, triple, and home run respectively [26] and 𝒫(1B|b),𝒫(2B|b),𝒫(3B|b), and 𝒫(HR|b) are defined using a machine learning model that takes as input the spray angle, launch angle, exit speed, and batter handedness and returns the probability of each hit type conditioned on the batted ball not being an out.

### B. OptimusPitch

OptimusPitch (OP) is a neural network developed by [18] for the pitching game. It is used to estimate $𝒫(x|\pi_{o},\pi_{u},\tau ,c)$ for all pitch outcomes x∈𝒳 in the expected run value functions (5) and (10). The original version of OP was a recurrent neural network that considered the full prior sequence of pitches and their outcomes when predicting the outcome of the next pitch. That version would have made calculating the complete defensive strategy impractical because the strategy for the next pitch cannot be calculated until the outcome of the current pitch is known. Thus, we created a simpler version of OP that just takes as input the release speed, movement, spin rate, and location of the current pitch along with the batter and pitcher handedness, the balls and strikes, and a batter-specific vector embedding. The batter embedding is similar to the embeddings used in the density neural nets and ensures that the pitching strategies are batter-specific. Our simpler version of OP makes well-calibrated predictions on the pitch outcomes, as evidenced by the calibration plots on a validation dataset (Fig 17), so we are confident in the estimated pitch outcome probabilities and therefore the expected runs utility function.

**Fig 17:**
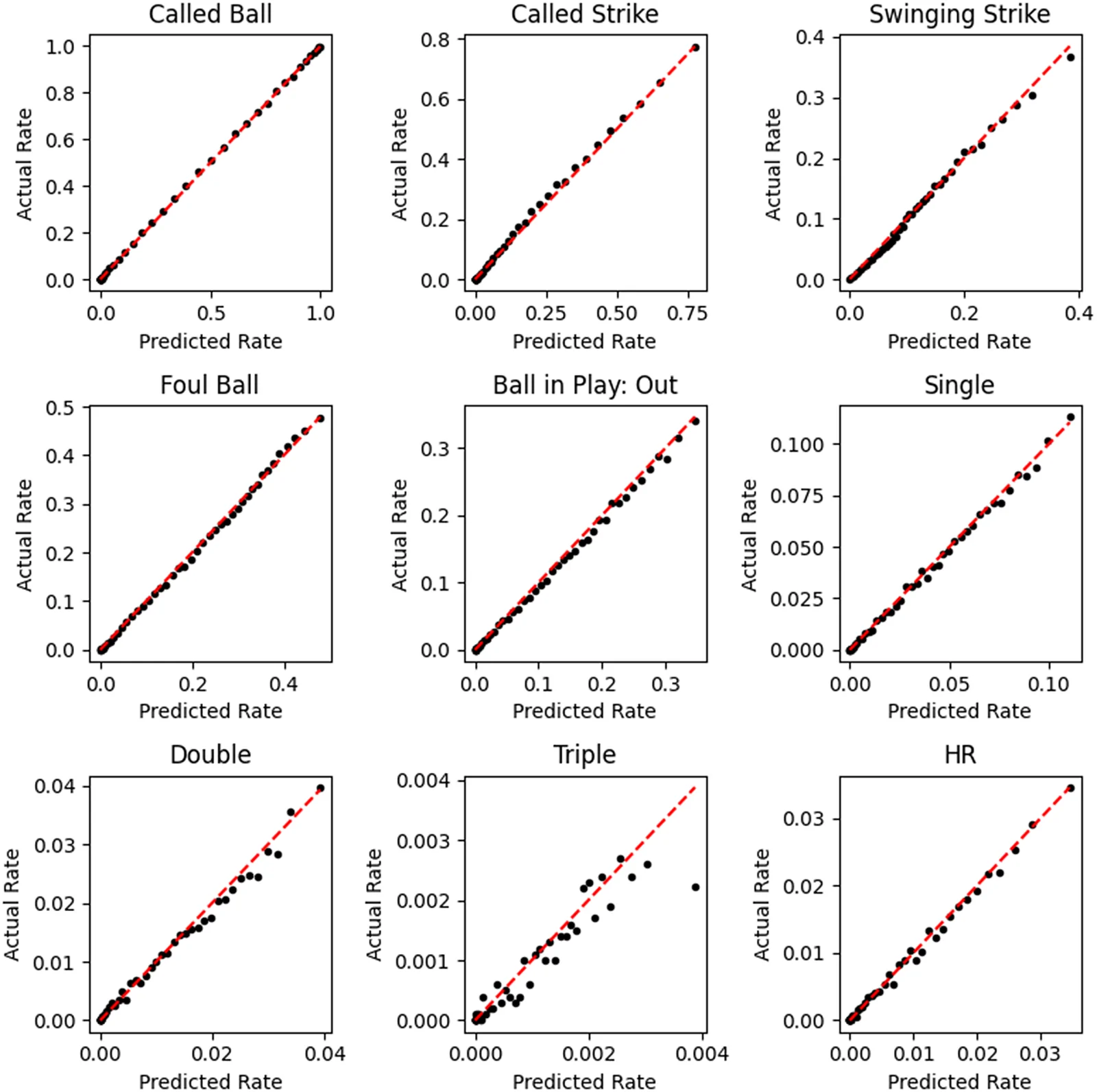
OptimusPitch probability calibration plots.

### C. Pitcher execution error distributions

To estimate the execution error distributions for individual pitchers, we utilize the *Joint Estimation of Execution and Decision-making Skill* (JEEDS) method [27]. The JEEDS algorithm takes as input the observed actions of an agent acting in a domain, and produces as output an estimate of the execution error distribution (execution skill) and a parameter representing the agent’s decision-making skill. Crucially, JEEDS does not require knowledge of the action the agent intended, only the one that actually was executed. JEEDS also needs to know the reward that the agent would get for any action that could be executed.

In our case each observed action is a pitch from a pitcher. To estimate pitcher accuracy we specifically focus on the location where the pitch crosses the plate. Using OptimusPitch (OP) (see Appendix B) combined with an expected run value function, the reward for any possible location the pitcher could have located the observed pitch is computed. This is done by modifying the pitch location while keeping the other characteristics of the pitch (velocity, movement, spin, ...) fixed, and then passing the modified pitch data into OP and the expected run function, resulting in a single value estimated for that location. This constitutes the reward information required by JEEDS. In our work JEEDS produced an execution error estimate for each pitcher and pitch type using up to 1000 most recent pitches of that type available for each pitcher. The estimate produced by JEEDS is the standard deviation of a symmetric two-dimensional multivariate Gaussian distribution. This distribution, centered on an intended target location, is used as the distribution over actual pitch locations required elsewhere in this work. The other pitch characteristics are assumed to be the pitcher’s average for each pitch type.
